# Comparative analysis of homologous aminopeptidase PepN from pathogenic and non-pathogenic mycobacteria reveals divergent traits

**DOI:** 10.1371/journal.pone.0215123

**Published:** 2019-04-10

**Authors:** Nishant Sharma, Suruchi Aggarwal, Saravanan Kumar, Rahul Sharma, Konika Choudhury, Niti Singh, Praapti Jayaswal, Renu Goel, Saima Wajid, Amit Kumar Yadav, Krishnamohan Atmakuri

**Affiliations:** 1 Vaccine and Infectious Disease Research Center, Translational Health Science and Technology Institute, Faridabad, Haryana, INDIA; 2 Drug Discovery Research Center, Translational Health Science and Technology Institute, Faridabad, Haryana, INDIA; 3 Proteomics Facility, Thermo Fisher Scientific Pvt. Ltd., Bengaluru, Karnataka, INDIA; 4 INDIAManipal University, Manipal, Karnataka, INDIA; 5 Dept. of Biotechnology, Jamia Hamdard, New Delhi; Cornell University, UNITED STATES

## Abstract

*Mycobacterium tuberculosis* (Mtb) secretes proteases and peptidases to subjugate its host. Out of its sixty plus proteases, atleast three are reported to reach host macrophages. In this study, we show that Mtb also delivers a lysyl alanine aminopeptidase, PepN (Rv2467) into host macrophage cytosol. Our comparative *in silico* analysis shows PepN_Mtb_ highly conserved across all pathogenic mycobacteria. Non-pathogenic mycobacteria including *M*. *smegmatis* (Msm) also encode *pepN*. PepN protein levels in both Mtb (pathogenic) and Msm (non-pathogenic) remain uniform across all *in vitro* growth phases. Despite such tight maintenance of PepNs’ steady state levels, upon supplementation, Mtb alone allows accumulation of any excessive PepN. In contrast, Msm does not. It not only proteolyzes, but also secretes out the excessive PepN, be it native or foreign. Interestingly, while PepN_Mtb_ is required for modulating virulence in *vivo*, PepN_Msm_ is essential for Msm growth *in vitro*. Despite such essentiality difference, both PepN_Mtb_ and PepN_Msm_ harbor almost identical N-terminal M1-type peptidase domains that significantly align in their amino acid sequences and overlap in their secondary structures. Their C-terminal ERAP1_C-like domains however align much more moderately. Our *in vitro* macrophage-based infection experiments with MtbΔ*pepN*-expressing *pepN*_Msm_ reveals PepN_Msm_ also retaining the ability to reach host cytosol. Lastly, but notably, we determined the PepN_Mtb_ and PepN_Msm_ interactomes and found them to barely coincide. While PepN_Mtb_ chiefly interacts with Mtb’s secreted proteins, PepN_Msm_ primarily coimmunoprecipitates with Msm’s housekeeping proteins. Thus, despite high sequence homology and several common properties, our comparative analytical study reveals host-centric traits of pathogenic and bacterial-centric traits of non-pathogenic PepNs.

## Introduction

Annually, worldwide, atleast a million people die of Tuberculosis (TB) [1]. To establish infection and hijack its host, *Mycobacterium tuberculosis* (Mtb) injects a battery of arsenal [2–5]. Mtb’s stockpile is predicted to include lipids, proteins, sugars and small molecules. Over the years, though several aspects of the Mtb’s biology have been discovered, to this day, only few of Mtb’s effectors that manipulate host cellular processes have been identified and their roles determined [2–6]. For example, SapM is a secreted lipid phosphatase that prevents phagosome-lysosome fusion [6]. ESAT-6 is an early secretory antigenic target protein that induces apoptosis [7], inhibits generation of reactive oxygen species [8] and suppresses antigen presentation by MHC1 [9]. ManLAM is a mannose-capped Lipoarabinomannan that also inhibits phagosome-lysosome fusion and T-cell receptor-mediated signaling [10].

Often, bacterial pathogens exploit their proteases and peptidases to target host-specific functions [11,12]. Mtb encodes sixty plus proteases and peptidases [13]. Among them, Zmp1, Msh1 and Rv3668c are known to access host macrophage cytosol. Zmp1 is a Zinc metalloprotease that inactivates inflammasome and arrests phagosome maturation [14]. Msh1 (Rv2672) is a protease that aids the pathogen to utilize host lipids, especially during hypoxic conditions [15]. Rv3668c is a serine protease that modulates inflammatory responses of the host [16].

Thus far, no literature exists on Mtb-encoded peptidases directly accessing host macrophages. However, in *in-vitro* lab cultures, few Mtb aminopeptidases atleast reach spent media (SM). For example, MapB (Rv2861c), an Iron-binding metallo-L-methionyl aminopeptidase that helps remove L-Methionine from selective nascent Mtb proteins reaches SM [17]. Similarly, PepC (Rv0800), a predicted aminopeptidase reaches SM [4], but its specific functions are yet undetermined.

PepN_Mtb_ (Rv2467) is a secreted [4], M1 family zinc metallo-aminopeptidase speculated to cleave proteins at their lysines, alanines, arginines and leucines [18]. Similar to other M1 family members, it also harbors a classical ‘GXMEN’ peptidase active site, and a HEXXH-(X18)-E zinc-binding motif (https://pfam.xfam.org/protein/L7N655) [18,19]. Most M1 members play pivotal roles in survival, cell maintenance, growth and development, virulence and pathogenesis [11]. They also are good vaccine antigens [20]. Though PepN role in Mtb is still unknown, a transposon insertion into its open reading frame aided Mtb to rapidly kill SCID mice *in vivo* but did not influence Mtb’s growth *in vitro* [21–23]. In a more recent study, insertion of Himar1 transposon into *pepN* resulted in H37Rv attenuation in wild-type mice [24].

PepN is also encoded by non-pathogenic mycobacteria including *M*. *smegmatis* (Msm). It (MSMEG_4690) also harbors an M1 peptidase domain including the GXMEN and HEXXH motifs (https://pfam.xfam.org/protein/A0R1B3) [19]. However, thus far, no traits of PepN_Msm_ have been reported. Here, using both *in silico* and *in vitro* approaches, we compared PepNs from both pathogenic (Mtb, H37Rv) and non-pathogenic (Msm, mc^2^155) mycobacteria to find common and distinct traits. Identification of their distinct traits also helped us predict their possible roles.

## Material and methods

### Bacterial strains and growth conditions

*Mycobacterium tuberculosis*, H37Rv (Mtb) was grown *in vitro* at 37°C in Middlebrook 7H9 broth (Thermo Fisher Scientific, USA) or 7H11 Middlebrook agar (Thermo Fisher Scientific, USA) supplemented with 10% Oleic acid-Bovine Albumin fraction V-Dextrose-Catalase (1X OADC), 0.2% (for broth)/0.5% (for agar) glycerol and 0.05% Tween 80. *Mycobacterium smegmatis*, mc^2^155 (Msm) was grown *in vitro* at 37°C in Middlebrook 7H9 broth or 7H11 Middlebrook agar supplemented with 10% Bovine Albumin fraction V-Dextrose-Catalase (1X ADC), 0.2% (for broth)/0.5% (for agar) glycerol and 0.05% Tween 80 [25].

*Escherichia coli* (*E*. *coli*) DH5α (Thermo Fisher Scientific, USA) was used for routine cloning and grown in Luria-Bertani (LB) broth/agar-based medium (Thermo Fisher Scientific, USA) at 37°C [26]. For recombinant protein expression, *E*. *coli* C41(DE3) was used [27]. For Gateway cloning, donor and destination vectors were maintained in *E*. *coli* DB3.1 (Thermo Fisher Scientific, USA).

Where necessary, to maintain plasmids, we used the following final concentrations of antibiotics: For (i) Mtb and Msm–Hygromycin, 50 μg/ml and Kanamycin, 25 μg/ml; and (ii) *E*. *coli*–Chloramphenicol, 25 μg/ml, Hygromycin, 150 μg/ml, and Kanamycin, 100 μg/ml.

### Molecular cloning

Employing standard restriction enzymes and/or Gateway cloning system (Thermo Fisher Scientific, USA), we generated the required plasmids (S1 Table). Derivatives of pDONR221 were generated and used as Gateway entry vectors. All expression constructs were derivatives of Tetracycline- (Tet) inducible, Gateway destination plasmid pTetSG [25] (kind gift of Dr. Sarah Fortune). Gateway cloning was performed as per manufacturer’s recommendations (Thermo Fisher Scientific, USA). To generate knockout, suicidal plasmid pJM1 (kind gift of Dr. Chris Sassetti) was first modified to insert required multiple cloning sites (pKA1) and then employed as base vector to clone both upstream and downstream flanking regions to *pepN*. To generate recombinant PepN_Mtb_ for antibody generation, an expression vector pET28a (Merck, USA) [28] was used. To generate *pepN*_Msm_::*ssrA*, using KAP447, KAP 469 and KAP470, we PCR amplified 1130 bp fragment from 3’ end of *pepN*_Msm_ and cloned the fusion (3’-*pepN*_Msm_::*ssrA*) into pKA2 to generate pNS38. Plasmid pNS39 is a derivative of pNS38 that lacks the ssrA tag. Plasmids and primers used/generated are listed as S1 and S2 Tables respectively.

Plasmid DNA from DH5α was extracted using pDNA miniprep kit (MDI, India) as per manufacturer recommendations. Msm and Mtb genomic DNA and total RNA were extracted as per recommended protocols [29]. PCRs were performed using high fidelity Phusion or Q5 DNA polymerases (New England Biolabs, USA) in Vapo-protect ProS Mastercycler Systems (Eppendorf, Germany). Amplicons obtained were electrophoresed on 0.8% agarose gel and eluted using Gel extraction kit (MDI). DNA ligations were performed using T4 DNA ligase—Quick Ligation kit (New England Biolabs, USA).

### Bioinformatics tools for comparative analysis

Employing CLUSTALW, a multiprotein sequence alignment tool [30], we aligned PepN_Mtb_ from Mtb—H37Rv (UniProt entry L7N655), and PepN_Msm_ from Msm—mc^2^155 (UniProt entry A0R1B3) and determined their percentage identity, similarity and differences. To identify significant alignment hits, we employed BLAST P [31] and SMARTBLAST local alignment search tools and separately queried with PepN_Mtb_ and PepN_Msm_. To determine possible differences in the PepNs from pathogenic and non-pathogenic mycobacteria, we employed Expresso [32], a T-COFFEE flavor that aligns multiple protein sequences using structural information. To align all query sequences structurally, Expresso compares each queried sequence to its closest protein crystal structures in PDB archive. Narrowing on identified structure(s) as reference, it aligns multiple sequences structurally [32]. We aligned PepN_Mtb_ (UniProt entry L7N655), its orthologues viz. *M*. *bovis* (UniProt entry Q7TYI7; NCBI Reference Sequence: WP_047709652.1), *M*. *caprae* (NCBI Reference Sequence: WP_075744548.1), *M*. *africanum* (NCBI Reference Sequence: WP_031669696.1), *M*. *canetti* (NCBI Reference Sequence: WP_015303505.1), *M*. *leprae* (UniProt entry Q9CBX9), all from Mtb complex and finally PepN_Msm_ (UniProt entry A0R1B3). ‘Good’, ‘average’ and ‘bad’ are algorithm outputs displayed by Expresso to indicate high, medium and poor levels of structure-based sequence homology.

### Transformation of Msm and Mtb

Msm and Mtb were transformed as per standard protocol [29]. Briefly, using a GenePulser Xcell (Bio-Rad, USA), plasmid DNA (100/300 ng for Msm and Mtb respectively) was used to transform freshly made Msm/Mtb electrocompetent cells. Plasmid DNA and required volume of electrocompetent cells were mixed, then transferred to a fresh, 2 mm, sterile electrocell/cuvette and pulsed at 2.5kV, 25uF and 1000 Ohms. Electroporated cell mixture was immediately recovered in three ml of Middlebrook 7H9 broth (supplemented with 1X OADC/ADC with 0.2% Glycerol and 0.05% Tween 80) by incubating for 3/24 h (Msm/Mtb respectively) at 37°C and 200 rpm. Cells were pellet down at RT, 3000 RPM, resuspended in 100 μl of fresh 7H9 broth and plated on 7H11 agar supplemented with ADC/OADC and appropriate antibiotics (when necessary) and transformants selected.

### Generation of MtbΔ*pepN* knockout and its complementation

To generate MtbΔ*pepN*, we employed homologous recombination-based gene knockout strategy using SacB as counter selection marker [33]. To avoid polar effect on Rv2466c (*pepN*’s flanking gene), we retained 324 base pairs (bp) from 5’ start of Rv2467 ORF and 24 bp at its 3’ end. The retained portions harbor neither the peptidase active site domain nor the C-terminal ERAP1-C_like domain. We PCR-amplified one kb flanking regions to *pepN* using KAP307 & KAP308 (upstream) and KAP309 and KAP336 (downstream), digested them (with *Eco*RV & *Spe*I and *Xho*I & *Sph*I respectively), purified and cloned them into similarly digested pKA1 to obtain pNS22 such that they flank either sides of the Hygromycin (Hyg)/Chloramphenicol resistance cassette. We electroporated two μg of pNS22 into freshly prepared H37Rv electrocompetent cells [29] and selected transformants on 7H11 agar plates containing 1X OADC and Hyg 50 μg/ml. We screened the obtained transformants for sensitivity to sucrose. Those that grew on 10% sucrose turned out to be false positives for double crossover. Using 10 colonies verified for single crossover at the expected locus (no growth on 10% sucrose), we grew them in fresh 7H9 broth + 1X OADC and Hyg 50 ug/ml to an optical density (O.D. at A_600nm_) of 0.1 and spread plated them on 7H11 agar plates containing 1X OADC, Hyg 50 μg/ml and 10% sucrose. We PCR-confirmed few colonies that emerged for loss of *sacB* region. Then we PCR-confirmed them for double crossover and loss of *pepN*. We finally confirmed *pepN* deletion by Southern [34] and western analysis [35].

Using RvΔ*pepN*325-2562 bp (referred in the manuscript as RvΔ*pepN*) cells, we complemented them with either full length PepN_Mtb_, or its active site mutant (mPepN_Mtb_) or full length PepN_Msm_, all expressing under Tet-inducible promoter. We PCR-amplified full length *pepN*_Mtb_ with KAP8 and KAP11. Similarly, we PCR-amplified full length *pepN*_Msm_ with KAP317 and KAP319. To add *att*B1 and *att*B2 regions to their 5’ and 3’ end, we PCR-amplified the first round Gateway amplicons with KAP5 and KAP6. We recombined the amplicons separately with pDONR221 to obtain pDONR221+*pepN*_Mtb_ (pNS24) and pDONR221+*pepN*_Msm_ (pNS28). We then recombined each with pTetSG [25] to obtain pNS25 (pTetSG+*pepN*_Mtb_) and pNS29 (pTetSG+ *pepN*_Msm_).

### Southern analysis

Southern was performed as per standard protocols [34]. Briefly, around 4 μg of genomic DNA of WT Mtb and MtbΔ*pepN* was separately digested O/N with *Pvu*I and *Not*I. The digested genomic DNA was loaded onto 0.8% agarose gel and electrophoretically resolved. The gel was washed in autoclaved Milli-Q water, depurinated (0.2 N HCl, 10 min), washed twice with autoclaved Milli-Q water (5 min each), and denatured (1.5 M NaCl, 0.5 M NaOH for 45 min). Gel was rinsed for 10 min in autoclaved Milli-Q water, neutralized for 45 min with 1 M Ammonium acetate, washed for 10 min with autoclaved Milli-Q water and transferred O/N onto HyBond Nylon + membrane by capillary transfer with 10X SSPE buffer. After transfer, genomic DNA was UV cross-linked with energy of 120 mJ/cm^2^ (CL-1000 Ultraviolet Crosslinker, UVP, UK), prehybridized in hybridization bottles for 3 h at 42°C and probed overnight at 42°C with Digoxigenin (DIG) [8]—labelled probe in DIG Easy Hyb buffer in hybridization oven. The probe amplicon was generated with KAP8 and KAP474 and labelled as per manufacturer’s recommendations (Thermo Fisher Scientific, USA). The probed membrane was washed sequentially at 60°C twice each with 2X SSPE containing 0.1% SDS and 0.5X SSPE containing 0.1% SDS, then rinsed 15 min in washing buffer (0.1 M Maleic acid, 0.15M NaCl, pH 7.5, 0.3% Tween 20 (v/v)), blocked (1 h with blocking buffer diluted to ratio of 1:10 in Maleic acid buffer (0.1 M Maleic acid, 0.15M NaCl, pH 7.5), and incubated for 30 min with anti-DIG antibody at (1:10,000 in blocking buffer). The blot was then washed again 4 times in washing buffer (15 min each), equilibrated with detection buffer (0.1M Tris-HCl, 0.1 M NaCl, pH 9.5) for 5 min and developed with CSPD substrate and chemiluminescent signal monitored on the gel documentation system (BioRad, USA).

### Site-directed mutagenesis

To generate the double mutant of *pepN*_Mtb_ (m*pepN*_Mtb_; GAMEN to GAAAN and HEXXH to AAXXH), we performed site-directed mutagenesis as per Phusion site-directed mutagenesis kit recommendations (Thermo Fisher Scientific, USA). Briefly, using pDONR221+*pepN*_Mtb_ as template, KAP225 and KAP226 as primers and Phusion High-Fidelity polymerase (New England Biolabs, USA), we first mutated GAMEN to GAAAN the active site of M1 peptidase. Then, using the mutant clone (pNS30; confirmed by sequencing) and KAP227 and KAP228 as primers, we mutated HEXXH (zinc-binding motif) to AAXXH. We confirmed the double mutant (pNS32) by sequencing and recombined it to pTetSG [25] to obtain pNS35. As per manufacturer’s recommendations, we used *Dpn*I to digest off the template pDNA.

### Anti-PepN antibody generation

Using Mtb genomic DNA as template and KAP315 and KAP316 primers, *pepN*_Mtb_ was PCR amplified and cloned as a *Nde*I/*Sac*I fragment into similarly digested pET28a to obtain 6X-His::*pepN* (pNS23; S1 Table). C41 DE3 (pLysS) *E*. *coli* strain harboring 6X-His::*pepN* was induced with 1 mM Isopropyl β-D-1-thiogalactopyranoside (IPTG) for 16 h at 37°C as per standard protocols [28]. The induced culture was spun down at 4°C and 10,000 RPM. The washed pellet was boiled in 1X Laemmli buffer for 15 min at 95°C and loaded on 10% SDS-PAGE to evaluate for over-expression. Since, both HisPur Cobalt and Ni-NTA beads retained several contaminating non-specific proteins, we cut the overexpressed band out, eluted proteins and generated polyclonal antibodies to the mixture in rabbits (Link biotech, India). The specificity of the generated antibody (Ab) was verified by western analysis [35] to whole cell protein lysates of Msm and Mtb. The antisera was further purified as in [36] and eluate immediately neutralized with drops of 1 M Tris, pH 7.5 and stored with 0.1% BSA (Bio Basic, Canada) and 0.02% Sodium azide (Sigma-Aldrich, USA) for future use.

### RNA extraction and RT-PCR

Approx. 2 x 10^9^ Mtb and Msm cells were used for RNA isolation. RNA was isolated by DNA, RNA and Protein purification Kit as per manufacturer (Machery-Nagel, Germany) protocol. Briefly, cell pellets were resuspended in 200 μl of TE (10 mM Tris-HCl, 1 mM EDTA, pH 8.0) with lysozyme (2 mg/ml) and lysed in the supplied buffer RA1 with β-ME (1:1000). The lysates were then passed through supplied purple colored nucleospin columns. The elutes were mixed with 70% ethanol and the mixtures passed through RNA-binding (blue colored) nucleospin columns. The bound RNA was desalted, treated with DNase, washed and eluted as per manufacturer’s recommendations. Three μg of extracted RNA was treated with 1 μl of Turbo DNase (Turbo DNA-free Kit—Thermo Fischer Scientific, USA) and genomic DNA contamination eliminated as per manufacturer’s protocol. Two μg of the extracted RNA was used to generate cDNA with PrimeScript 1^st^ strand cDNA synthesis Kit (Clonetech, Takara, Japan) as per manufacturer’s recommendations. Equal volume (1 μl) of generated cDNA was used to set up qRT-PCR with 5X HOT FIREPol Evagreen qPCR Mix Plus (SYBR Green; Solis Biodyne, Estonia) on Stratagene mx3005p system (Agilent technologies, USA). Primer pairs KAP411, KAP412 (for Mtb) and KAP413, KAP414 (for Msm) were used for qRT-PCR (S2 Table). The Ct values were normalized with *sigA* and *mysA* [29] as controls for Mtb and Msm respectively. The *sigA* was amplified with primer pair KAP463 and KAP464. The *mysA* were amplified with primer pair KAP464 and KAP465. The sequence of primers used are listed in S2 Table

### Co-immunoprecipitation assay and interactomes identification

Mid-log cultures of Mtb and Msm were pellet down at 4000 RPM for 10 min, pellets washed and lysed in bead-beating buffer (1 mM Tris (pH 6.8), with 0.5 mM EDTA). Bead beating was performed for 10 cycles, 30 s each, with 1 min incubation on ice between each cycle (Biospec Products, USA). The lysate was filtered through 0.22 μM disc filter (MDI, India) and protein estimated using BCA protein estimation kit (Thermo Fisher Scientific, USA). One mg each of Mtb and Msm lysate were separately incubated on a gentle rocker (30 cycles/min), each with 100 μg of purified anti-PepN antibody for 16 h at 4°C. The protein complexes were pulled down by further incubating the lysates with 75 μl of SureBeads Protein G Magnetic Beads (BioRad, USA) for 2 h at 4°C. As negative controls, the bacterial lysates were co-incubated with SureBeads alone or SureBeads together with pre-immune sera. Beads were washed in 1X TBST as per manufacturer’s protocol and eluted in 100 μl 7.5 M Urea (2 h at 25°C with frequent gentle mix) and processed for mass spectrometry (MS).

To subtract out the non-specifically bound lysate proteins from PepN_Mtb_ and PepN_Msm_ interactomes, we simultaneously performed co-immunoprecipitation on same lysates with beads alone and beads with pre-immune sera. To eliminate the non-specifically bound proteins further, in Mtb, we also performed similar pulldowns with lysate of *pepN* KO. To further shortlist PepN_Mtb_ interactome, we also co-immunoprecipitated proteins with lysates of *pepN* KO complemented with either wild-type PepN_Mtb_ or mPepN_Mtb_. All co-immunoprecipitations were performed as per manufacturer (BioRad, USA) recommendations and precipitated pellets were again processed by MS to identify PepN interacting proteins. Technical triplicates of each sample were run on Nano-LC Q-Exactive Plus Orbitrap MS (Thermo Fisher Scientific, USA), MS data collected and analyzed to identify potential interactomes to PepN_Mtb_ and PepN_Msm_. For every sample, only proteins that were common in at least two replicates with more than one unique peptide were unified to generate a single protein list and the quantitative measurements were averaged using geometric mean. The final protein lists thus obtained were used for comparative analyses to finally generate S3 and S4 Tables.

To shortlist PepN_Mtb_ interactome, we first combined protein lists obtained from (a) wild-type Mtb lysate co-immunoprecipitated with (i) no antibody (NoAb_Mtb_) & (ii) with pre-immune sera (PI_Mtb_); and (b) *pepN*_Mtb_ KO lysate co-immunoprecipitated with anti-PepN antibodies (KOAb). This combined list includes all non-specifically co-immunoprecipitated Mtb and *pepN*_Mtb_ KO lysate proteins. As expected, there was no PepN in this list. To generate the interactome of PepN_Mtb_, all proteins from the above combined list (NoAb_Mtb_ + PI_Mtb_ + KOAb) were subtracted out from individual protein lists of (a) wild-type Mtb lysate co-immunoprecipitated with anti-PepN antibodies (WT_Mtb_Ab) and (b) lysates of *pepN*_Mtb_ KO complimented with either *pepN*_Mtb_ or m*pepN*_Mtb_, (KOC_wt_Ab and KOC_m_Ab respectively) both co-immunoprecipitated with anti-PepN antibodies. The proteins thus finally shortlisted included those found common to (i) WT_Mtb_Ab & KOC_wt_Ab & KOC_m_Ab (36 proteins); (ii) KOC_wt_Ab and KOC_m_Ab (31 proteins); (iii) WT_Mtb_Ab & KOC_m_Ab (20 proteins) and (iv) unique to KOC_m_Ab. Thus, PepN_Mtb_ interactome finally constitutes 115 Mtb proteins (S4 Table).

Similarly, we first combined the protein lists of (a) wild-type Msm lysate co-immunoprecipitated with (i) no antibody (NoAb_Msm_) & (ii) with pre-immune sera (PI_Msm_) to generate the non-specifically co-immunoprecipitated Msm lysate proteins. This combined list was then subtracted out from protein list obtained by co-immunoprecipitating wild-type Msm lysate with anti-PepN antibodies (WT_Msm_Ab) to finally generate a list of 108 Msm proteins that formed the interactome of PepN_Msm_ (S3 Table).

To dissect these lists further, we first analyzed both protein lists for gene ontology (GO)/biological processes using Uniprot [37], and Mycobrowser [18]. Then, we broadly classified them into different functional groups manually merging similar functions.

Final interactome lists (S3 and S4 Tables) were analyzed for gene ontology (GO)/biological processes using Uniprot [37], and Mycobrowser [18].

### MS analysis

Trypsinization and peptides elution was as per manufacturer’s recommendations (Thermo Fisher Scientific, USA). Briefly, trypsinized samples was spun down at 10,000 RPM, 4°C for 20 min to remove debris and undissolved pellet. The clear supernatant was passed thrice through C18 columns. The unbound peptides were washed away with 0.1% FA and bound peptides eluted in 0.1% FA with 50% ACN. The eluates were vacuum dried and subjected to MS. The raw data obtained from mass spectrometry proteomics (for secretion and co-immunoprecipitation) studies has been submitted to public data repositories ProteomeXchange via Massive (http://massive.ucsd.edu/ProteoSAFe/static/massive.jsp) and PRIDE (http://www.ebi.ac.uk/pride/archive/. The mass spectrometry proteomics data for secretion (Table 1) can be downloaded from https://massive.ucsd.edu/ProteoSAFe/dataset.jsp?task=a5fa419b81a9436b940d736b4db03648. The data further submitted to PRIDE can be downloaded from ProteomeXchange (http://proteomecentral.proteomexchange.org). ProteomeXchange, MassIVE ID: MSV000081967; ProteomeXchange, PRIDE ID PXD008790.

**Table 1:** Upon overexpression of PepN, Msm secretes excess PepN into spent media.

SL No.	SAMPLES	Spectral counts (Number of peptides)*			
		Wild type		Overexpressed	
		PepN	EsxB^#^	PepN	EsxB^#^
1.	Mtb lysate– 1	**177**(20)	**58**(13)	**1924**(163)	**46**(14)
2.	Mtb lysate– 2	**250**(21)	**102**(25)	**702**(101)	**77**(20)
3.	Mtb culture supernatant—1	**285**(20)	**3777**(662)	**294**(10)	**2225**(460)
4.	Mtb culture supernatant—2	**378**(20)	**2514**(241)	**150**(6)	**1383**(191)
5.	Msm lysate– 1	**246**(18)	**15**(2)	**2497**(393)	**51**(11)
6.	Msm lysate– 2	**347**(15)	**29**(5)	**1574**(252)	**60**(7)
7.	Msm culture supernatant—1	**394**(15)	**499**(102)	**603**(61)	**183**(45)
8.	Msm culture supernatant—2	**123**(23)	**259**(28)	**588**(62)	**143**(10)

*peptides with 95% confidence

^#^[14]

*In vitro* grown mid-log cultures of wild-type Mtb, Mtb overexpressing PepN_Mtb_, wild-type Msm and Msm overexpressing PepN_Msm_ were pelleted down, pellets lysed and lysates stored. The spent media was filtered (0.22 μm filters) twice, culture supernatant proteins precipitated (5% TCA), washed and stored. Equal amount of total proteins from all lysates and culture supernatants were processed for MS (Materials and methods). 1 and 2 numericals in the samples column represent biological duplicates.

The data obtained from interactome proteomics (co-immunoprecipitation experiments) can be downloaded from ProteomeXchange (http://proteomecentral.proteomexchange.org). ProteomeXchange, PRIDE ID PXD009164.

### Trypsinization and peptides processing for MS analysis

Trypsinization and peptides elution was as per manufacturer’s recommendations (Thermo Fisher Scientific, USA). Briefly, known amount of protein (BCA, Thermo Fisher Scientific, USA) was resuspended in 50 mM Ammonium carbonate pH 8.0 containing 7.5 M Urea and 10 mM Dithiothreitol (DTT). After 1 h incubation at RT, Iodoacetamide was added to a final of 50 mM, gentle vortexed, and mixture incubated in dark for 1 h at room temperature (RT). Then DTT was added to a final of 35 mM and incubated in dark for 1 h at RT. Urea in the sample was then diluted to 0.5 M with 50 mM Ammonium carbonate containing 1 mM CaCl_2_ (pH 7.6). One μg (per 50 μg protein) of MS grade trypsin (Promega, USA) was added and samples incubated at 37°C for 16 h. Trypsinization was stopped by reducing pH to ~3 with formic acid (FA). The samples were lyophilized, resuspended in 0.1% FA (pH ~3) and purified using C18 spin columns (Thermo fisher Scientific, USA) as per manufacturer’s protocol.

Briefly, before adding samples to C18 columns, columns were spun at 3000 RPM for 1 min and sequentially washed thrice with 50% MS-grade Acetonitrile (ACN), 0.1% FA + 70% ACN and 0.1% FA. Trypsinized samples was spun down at 10,000 RPM, 4°C for 20 min to remove debris and undissolved pellet. The clear supernatant was passed thrice through C18 columns. The unbound peptides were washed away with 0.1% FA and bound peptides eluted in 0.1% FA with 50% ACN. The eluates were vacuum dried and subjected to MS.

### MS for interactome experiments

#### Nano-LC based reverse phase separation of tryptic peptides

Vacuum-dried tryptic peptide pellets were re-dissolved in 10 to 20 μL of 0.1% Formic acid (FA), vortexed gently and prior to injection centrifuged at 12000 RPM for 15 min. Around 3–5 μL of the sample was bound onto a pre-equilibrated Acclaim PepMap 100, 75 um x 2 cm, Nanoviper, C18 pre-column (Thermo Fisher Scientific, USA) at a flow rate of 4 μL/min. Reverse phase separation of the peptide mixture was performed using the Easy Nano-LC 1200 system (Thermo Fisher Scientific, USA) using a PepMap RSLC C18—Easy spray Analytical column of 75um x 50cm length at a flow rate of 250 to 300 nL/min with a solvent system comprising solvent A (0.1% FA), and solvent B (0.1%FA in 80% ACN, v/v). The column temperature was maintained at 40°C throughout the run. The gradient conditions were set to achieve 5 to 8% B for 2 min; followed by a linear increase of 8% to 20% B for 150 min, 20% to 40% B for 10 min, 40% to 80% B for 5 min, wash with 80% B for 5mins, 80% to 5% B for 2 min and 5% B for 6 min.

#### Q-Exactive Plus Orbitrap MS based protein identification

**Q-Exactive Plus MS instrument parameters.** Spectral measurements were attained using the Q-Exactive Plus Orbitrap MS platform (Thermo Fisher Scientific, USA) in the positive ion mode with an Electrospray voltage of 2.5 kV, Capillary temperature 300°C. For MS scan, at 70,000 resolution, the AGC target was set to achieve 3e6, IT-50ms, scan range 350 to 2000 m/z, Resolution-70,000 and for MS2 scan AGC was set to achieve 1e5, IT-100ms, Resolution-17,500. Samples were acquired using the TopN = 15—DDA (Data dependent acquisition) method and MS2 fragmentation was achieved through Higher Energy Collisional Dissociation (HCD) using a NCE (Normalized Collision Energy) value of 27. Singly charged, unassigned and >8 charged species were excluded from acquisition. Dynamic exclusion was set to 30 s, intensity threshold of 1.0e4, peptide match was set to preferred and Exclude isotopes—ON. Mass accuracy during the acquisition was ensured through the lock mass option using the Polysiloxane species (m/z 445.12003). The acquisition parameters were fed into the instrument using the Thermo X-Calibur software version 4.0 through the Tune Plus software interface version 2.8.

**Protein identification using the Proteome Discoverer software.** To generate protein identities, the raw data (.Raw) files were analyzed using Proteome Discoverer (PD; software version 2.1). Briefly, reference proteome database of *Mycobacterium tuberculosis* (strain ATCC 25618 / H37Rv) comprising 3,993 protein sequences (UP000001584) and *Mycobacterium smegmatis* (strain ATCC 700084 / mc^2^155) comprising 6,601 protein sequences (UP000000757) were used to achieve protein identities. Employing the processing and consensus workflow options available within the PD software, the Sequest search engine was used. Search parameters included MS tolerance: 10ppm, MS/MS tolerance: 0.02 Da, Enzyme specificity: Trypsin, Static modification: Carbamidomethylation (Cysteine), Dynamic modification: Methionine Oxidation, N-terminal acetylation, Maximum missed cleavage—2, only those protein entries with a FDR threshold of 0.01 were considered as identified in the current study. Identified proteins were exported for further in silico-based analysis.

### MS for PepN secretion experiments

All samples were analyzed by reverse-phase high-pressure liquid chromatography electrospray ionization tandem mass spectrometry using an Ekspert-nanoLC 415 system (Eksigent; Dublin, CA) which is directly connected to an ABSCIEX 5600 Triple-TOF (AB SCIEX; Concord, Canada) mass spectrometer, referred as Triple TOF system.

Reverse Phase -HPLC was performed via a trap and elute configuration using Ekspert-nanoLC 415 systemcolumns (Eksigent); the trap column (200 μm × 0.5 mm) and the analytical column (75 μm × 15 cm) were both manufacturer (Eksigent)-packed with 3 μm ChromXP C-18 (120 Å). Reverse-phase mobile phase consisted of mobile phase A: 2% acetonitrile/98% of 0.1% FA (v/v) in water, and mobile phase B: 98% acetonitrile/2% of 0.1% FA (v/v) in water. All samples were eluted from the analytical column at a flow rate of 250 nL/min using a initial gradient elution of 10% B from 0 to 5 min, transitioned to 40% over 120 min, ramping up to 90% B for 5 min, holding 90% B for 10 min, followed by re-equilibration of 2% B at 10 min with a total run time of 150 min. The analytical column temperature was maintained at 35°C to decrease retention time drift. The collected raw files spectra were stored in .wiff format. Autocalibration of spectra occurred after acquisition of every sample using dynamic LC–MS and MS/MS acquisitions of 100 fmol β-galactosidase.

Mass spectra and tandem mass spectra were recorded in positive-ion and “high-sensitivity” mode with a resolution of ~35,000 full-width half-maximum. Peptides were injected into the mass spectrometer using 10 μm SilicaTip electrospray PicoTip emitter (New Objective Cat. No. FS360-20-10-N-5-C7-CT), and the ion source was operated with the following parameters: ISVF = 2100; GS1 = 20; CUR = 25.

The data acquisition mode in DDA experiments was set to obtain a high resolution TOF-MS scan over a mass range 350–1250 m/z, followed by MS/MS scans of 20 ion candidates per cycle with activated rolling collision energy, operating the instrument in high sensitivity mode. The selection criteria for the parent ions included the intensity, where ions had to be greater than 150 cps, with a charge state between +2 to +5, mass tolerance of 50 mDa and were present on a dynamic exclusion list. Once an ion had been fragmented by MS/MS, its mass and isotopes were excluded from further MS/MS fragmentation for 12 s. Collision-induced dissociation was triggered by rolling collision energy. The ion accumulation time was set to 250 ms (MS) and to 70 ms (MS/MS).

### Database search

All DDA mass spectrometry files were searched in Protein Pilot software v. 5.0.1 (AB SCIEX) with the Paragon algorithm. For Paragon searches, the following settings were used: Sample type: Identification; Cysteine Alkylation: methyl methanethiosulfonate (MMTS), Digestion: Trypsin; Instrument: TripleTOF5600; Species: *Mycobacterium tuberculosis* (H37Rv) and *Mycobacterium smegmatis* (mc^2^155); Search effort: Thorough ID; Results Quality: 0.05. Only peptides with a confidence score of > 0.05 were considered for further analysis. The search was conducted using a through identification effort of a Swiss-Prot database from the UniProt website **(www.uniprot.org)**. False discovery rate analysis was also performed. Carbamidomethylation (C) was used as a fixed modification. The peptide and product ion tolerance of 0.05 Da was used for searches. The output of this search is a .group file and this file contains the following information that is required for targeted data extraction: protein name and UniProt accession, cleaved peptide sequence, modified peptide sequence, relative intensity, precursor charge, unused Protscore, confidence, and decoy result. The parameters used for identification of proteins includes: 1) Threshold of 1% accepted Global False discovery rate (G-FDR) proteins; 2) At least one unique peptide with 95% confidence. The false positive rates of the aforementioned filter criteria were all below 1%, estimated by using an individual reversed (decoy) sequence database.

### Mtb-mediated THP-1 infections and immunofluorescence

THP-1 (from Cell repository, NCCS, Pune, India; authenticated (on 25^th^ Nov 2016) by STR profiling at Lifecode Technologies Pvt. Ltd., INDIA; also verified by multiplex-PCR for no cross contamination with cell lines derived from Chinese hamster; grivet monkey, rat and mouse) were *in vitro*-cultured in RPMI-1640 medium containing 2 mM L-Glutamine, 10 mM HEPES (Thermo Fisher Scientific, USA), and supplemented with 10% fetal bovine serum (FBS), 0.05 mM β-Mercaptoethanol and 100 U/mL Penicillin-Streptomycin (all from Thermo Fisher Scientific, USA). THP-1 cells were seeded at 1x10^5^/well in 24-well plates on glass cover slip and differentiated before infection for 3 days at 37°C, 5% CO_2_ by adding 10 ng/ml Phorbol-12-myristate-13-acetate (PMA) (Sigma-Aldrich, USA). Differentiated-THP1 were used for infection and for immunofluorescence microscopic analysis.

Mtb-mediated infections of THP-1 were performed as described earlier [38]. Briefly, *in vitro* grown Mtb (~0.3 OD (A_600nm_)) were pellet down, washed in RPM1-1640 (having 2 mM L-Glutamine, 10 mM HEPES and 10% FBS) and filtered through 5μm syringe filters. Known number of Mtb cells were then added to differentiated THP-1 at an MOI of 1:10 and incubated for 4 h at 37°C, and 5% CO_2_. The uninfected controls and infected cells were washed thrice with 1X PBS containing Amikacin (200 μg/ml) and fresh RPM1-1640 (having 2 mM L-Glutamine, 10 mM HEPES, 10% FBS and 100U/ml of Penicillin-Streptomycin) added. Five days post-infection, cells were washed thrice with 1X PBS and fixed in 4% Para-formaldehyde. Before fixing, where necessary, Lysotracker green DND-26 (Thermo Fisher Scientific USA; final concentration: 50 nmol) was added to media and incubated for 1 h. The cells were washed thrice with 1X PBS and fixed in 4% Para-formaldehyde. Cells were blocked for 1 h with 1 X PBSAT buffer (1X PBS, pH 7.4 with 1% BSA, 0.5% Tween 20) and incubated for 1 h with primary antibody (anti-GRP94 antibody (Source–Rat; Abcam, USA) and anti-PepN antibody (This study; Source–Rabbit; both at 1:250 dilution in 1X PBSAT). The coverslips were washed thrice with 1X PBSAT and incubated for 1 h with appropriate secondary antibodies (Goat anti Rat for GRP94 and Goat anti-Rabbit for pepN) conjugated to either Alexa Flour 488 (1:500; for detection of GRP94) or Alexa Fluor 647 (1:500; for detection of PepN) (Southern Biotech, USA), washed thrice and mounted on slides with mounting media containing DAPI (Molecular Probes, Thermo Fisher Scientific, USA). Samples were then visualized under a 60X objective on a FV1000 Olympus Confocal microscope (Tokyo, Japan). The accompanying Olympus FV10-ASW version 2.01.03.10 software was used for image analysis.

## Results

In this study, we selected PepN_Mtb_ and PepN_Msm_ as representative PepNs of pathogenic and non-pathogenic mycobacteria respectively. We independently BLASTed (BLAST P) [31] them as query sequences and found PepN widely conserved across all mycobacterial species. We then aligned (CLUSTALW) [30] PepN_Mtb_ and PepN_Msm_ protein sequences for comparative analysis. Though, they align substantially well (~78% identity at amino acids level; 10.6084/m9.figshare.7873274), their N- and C-terminal end sequences show some divergence (10.6084/m9.figshare.7873274). To probe this divergence further, because of unavailability of their quaternary structures, employing Expresso [32], we aligned their secondary structures. Expresso aligns multiple protein sequences using structural information. Interestingly, while the N-terminal halves (Peptidase M1 N-terminal domain + Peptidase family M1 domain; https://pfam.xfam.org/protein/L7N655) of both pathogenic and non-pathogenic PepNs structurally aligned ‘good’ (S1 Fig), their C-terminal ERAP1_C-like domains aligned “average to bad” (S1 Fig; Good’, ‘average’ and ‘bad’ are algorithm outputs displayed by Expresso to indicate high, medium and poor levels of structure-based sequence homology). In contrast, both M1 peptidase and ERAP1_C-like domains of pathogenic PepNs (Mtb complex) structurally aligned ‘good’ throughout their lengths (10.6084/m9.figshare.7873442).

Interestingly, the ‘ERAP1’ of the C-terminal ERAP1_C-like domain refers to host Endoplasmic Reticulum (ER) aminopeptidase 1, also a M1 family member [39]. Given that both PepN and ERAP1 are M1 peptidases, we predicted that their quaternary structures would overlap. Using Coot [40], we overlapped the quaternary structure of human ERAP1 (3MDJ; 10.2210/pdb3MDJ/pdb) to the predicted quaternary structure of PepN_Mtb_ (homology-based protein modelling (Phyre2) [41] and found them to significantly resemble each other (rmsd value: 1.9A^o^) (10.6084/m9.figshare.7873451).

### Both PepN_Mtb_ and PepN_Msm_ primarily localize to host macrophage cytosol

**Fig 1:**
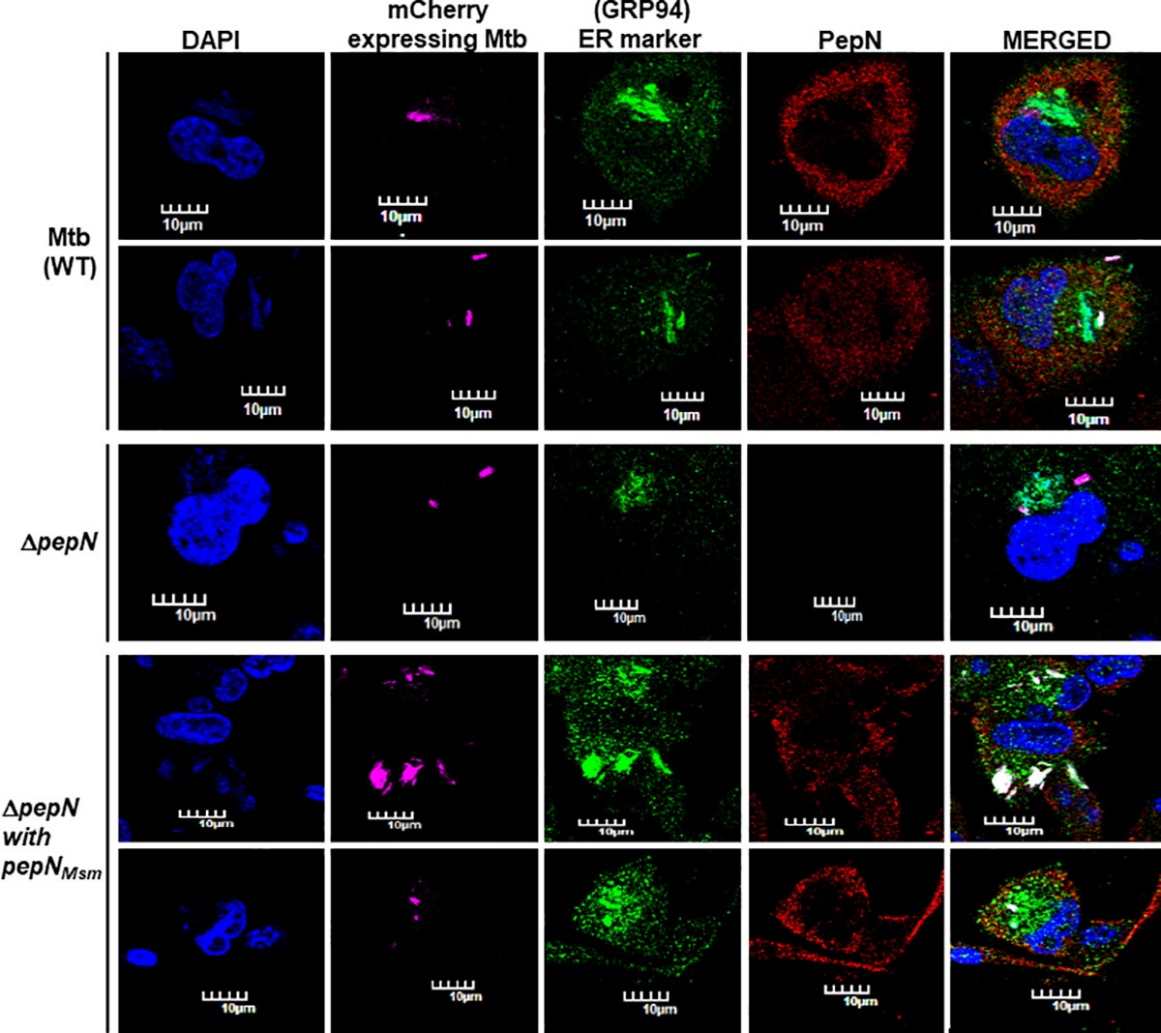
Both PepN_Mtb_ and PepN_Msm_ efficiently localize to host macrophage cytosol. Immunofluorescence-based localization analysis of PepN_Mtb_ in THP-1 infected with either WT Mtb (top two panel rows); MtbΔ*pepN* (middle (third) panel row) or MtbΔ*pepN* expressing *pepN*_Msm_ (bottom two panel rows). **Panel columns:** First: DAPI to track nuclei (blue); Second: virulent Mtb expressing mCherry (pink)—to locate infected THP-1; Third: ER-specific marker (green); Fourth: PepN (red); Final: merger of first four panels (of each row); Scale: 10 μm. Atleast 300 infected macrophages were visualized for confirming consistency of observed results. The immunofluorescence data is a representation of three independent experiments. Each independent experiment had biological duplicates.

Given PepN_Mtb_’s structurally similarity to host ERAP1, and PepN_Mtb_ presence in spent media (SM) of Mtb lab cultures [4], we wondered if Mtb delivers PepN_Mtb_ into host macrophages and if so specifically to ER. Interestingly, *insilico* analysis (LocSigDB) [42] of PepN_Mtb_ also indicated ER homing-like sequences spread across both domains (S2 Fig). Hence, we tested if upon infection into THP-1, a human macrophage-like cell line [38], Mtb delivers PepN_Mtb_ into ER of macrophages (Fig 1). We immunoflouresced Mtb-infected THP-1 with anti-PepN and anti-GRP94 (ER-specific marker) [43] antibodies. To our surprise, most of the secreted PepN_Mtb_ localized away from ER (Fig 1). Our nucleus-specific staining with DAPI also revealed that PepN_Mtb_ does not localize to THP-1’s nucleus (Fig 1). Using, Lysotracker Green, we determined that PepN_Mtb_ does not localize to lysosomes as well (S3 Fig). This absence of localization to ER, nucleus and lysosomes and the obtained localizing pattern (Fig 1) of PepN_Mtb_ indicate that most of the pathogen secreted PepN_Mtb_ localizes to THP-1 cytosol (Fig 1).

As expected, we did not see any PepN-specific signal in THP-1 infected with MtbΔ*pepN* (Fig 1). Since, PepN_Mtb_ and PepN_Msm_ exhibit high sequence homology (10.6084/m9.figshare.7873274) and structural identity (S1 Fig), we also tested if PepN_Msm_ harbors ER-homing signals and if so can reach ER. Again, *insilico* analysis (LocSigDB) [42] of PepN_Msm_ showed ER homing-like sequences spread across both domains (S4 Fig). MtbΔ*pepN* complemented with *pepN*_Msm_ was used for infecting THP-1. Similar to PepN_Mtb_, most of PepN_Msm_ also did not reach nucleus, ER (Fig 1) and lysosomes (S3 Fig). Thus, despite (i) harboring ERAP1_C-like domains; (ii) containing ER homing-like signals, and (iii) structurally resembling host ERAP1 (10.6084/m9.figshare.7873451), neither PepN_Mtb_ nor PepN_Msm_ localized to ER. Their absence to localize to ER, nucleus (Fig 1) and lysosomes (S3 Fig), the staining pattern observed, and by process of elimination, we infer that both PepN_Mtb_ and PepN_Msm_ primarily localize to host macrophage cytosol (Fig 1).

### PepN_Msm_ is necessary for *in vitro* growth of Msm

We speculated earlier that differences in PepN_Mtb_ and PepN_Msm_ amino acids sequence (10.6084/m9.figshare.7873274) and secondary structures (S1 Fig) might influence their localization in host cells. However, given their similar localization patterns (Fig 1), we wondered if any of the above said differences influence PepN_Mtb_ and PepN_Msm_ roles in their cognate parent mycobacterial environment.

To test this, employing standard homologous recombination-based gene knockout (KO) strategy [33], we set out to generate MtbΔ*pepN* and MsmΔ*pepN*. We effortlessly could knock out *pepN* from Mtb (H37Rv; S5 Fig) and hence could readily complement it (MtbΔ*pepN*) with *pepN*_Msm_ and determine PepN_Msm_ localization in THP-1 (Fig 1). Besides, MtbΔ*pepN* grew similar to WT-Mtb confirming its non-essentiality *in vitro*. In contrast, despite several attempts, we failed to generate MsmΔ*pepN*. Our attempts to conditionally KO the genomic copy (by episomally expressing *pepN*_Msm_ on an inducible promoter) also failed. To atleast obtain a knockdown phenotype, we adopted CRISPRi [44]. Though this knockdown strategy reduced *pepN*_Msm_ transcripts by ~80% (10.6084/m9.figshare.7873469), surprisingly, the steady state levels of PepN_Msm_ remained unaltered (10.6084/m9.figshare.7873469). This suggested that either PepN_Msm_ has an extended half-life or its levels are strictly maintained in Msm.

**Fig 2:**
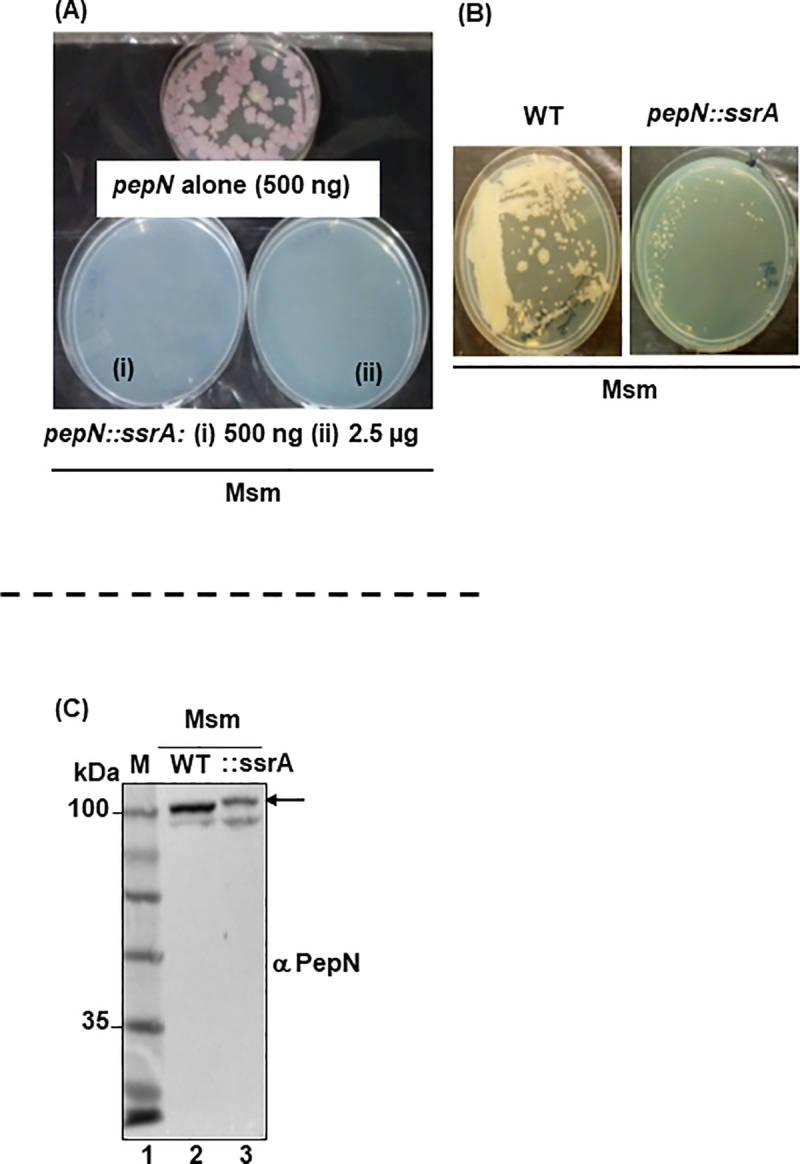
PepN_Msm_ is necessary for Msm’s *in vitro* growth. **(A)** Freshly prepared electrocompetent Msm were transformed with suicidal vector containing 3’ end (~ 1 kb) of *pepN* alone (pNS39; top single plate; 500 ng pDNA) or *pepN* fused at its 3’ end with degradation tag *ssrA* (pNS38; i—500 ng; and ii—2500 ng of pDNA) and plated on Kanamycin (25 μg/ml) containing 7H11 + ADC plates. Similar results were observed with three independent rounds of electroporation. Transformants with pNS39 emerged in 5–6 days post transformation as it is a suicidal plasmid. Top plate was incubated for additional 36–48 h to see robust growth of colonies that emerged. The bottom plates (i) and (ii) were photographed after 3 weeks of incubation. **(B)** To compare growth rate, equal number (~10,000 bacteria) of WT Msm and possible revertant Msm expressing *pepN*::*ssrA* were spotted and then streaked on 7H11 + ADC plates. Both plates did not contain Hygromycin so that the speed of growth could be compared. The image recorded is after 4 days of incubation at 37°C. Broth cultures could not be used for comparison as the Msm expressing *pepN*::*ssrA* clumps in broth. **(C)** Equal amount of total proteins from WT Msm and Msm expression *pepN*::*ssrA* (revertant) was resolved on 10% SDS-PAGE gels, proteins transferred onto nitrocellulose membrane and western analysis performed with anti-PepN antibody (1:2500). M–protein marker. The major form of PepN_Msm_ (arrow) in the potential Msm revertant exhibit increase in size because of degradation tag (SsrA) fusion to PepN.

To test this, to 3’ end of *pepN*_Msm_, just before its stop codon, we genetically fused *ssrA*, the canonical protein degradation signal [45]. We expected that SsrA-mediated depletion of PepN_Msm_::SsrA might generate atleast a knockdown phenotype. Despite repeated attempts, to our surprise, no transformants emerged even after incubation of plates for three weeks. When we used a similar construct lacking just the *ssrA* tag, transformants emerged within 6–7 d (Fig 2A). During one of several such attempts, in the 4th week, only one colony, a potential revertant (to PepN_Msm_ knock down) emerged that grew very slowly (than WT Msm; Fig 2B). This colony did not harbor any compensatory mutation in its entire *pepN*_Msm_::*ssrA* length as its amino acid sequence was identical to WT PepN. Western analysis also showed PepN_Msm_::ssrA fusion protein moving at the expected molecular weight (Fig 2C). Though we are yet to determine the location of the compensatory mutation, these above observations clearly suggest that PepN_Msm_ is possibly necessary for *in vitro* growth of Msm. In contrast, Mtb’s PepN is unnecessary for Mtb growth *in vitro*.

### Msm and not Mtb proteolyzes excess of its own PepN

**Fig 3:**
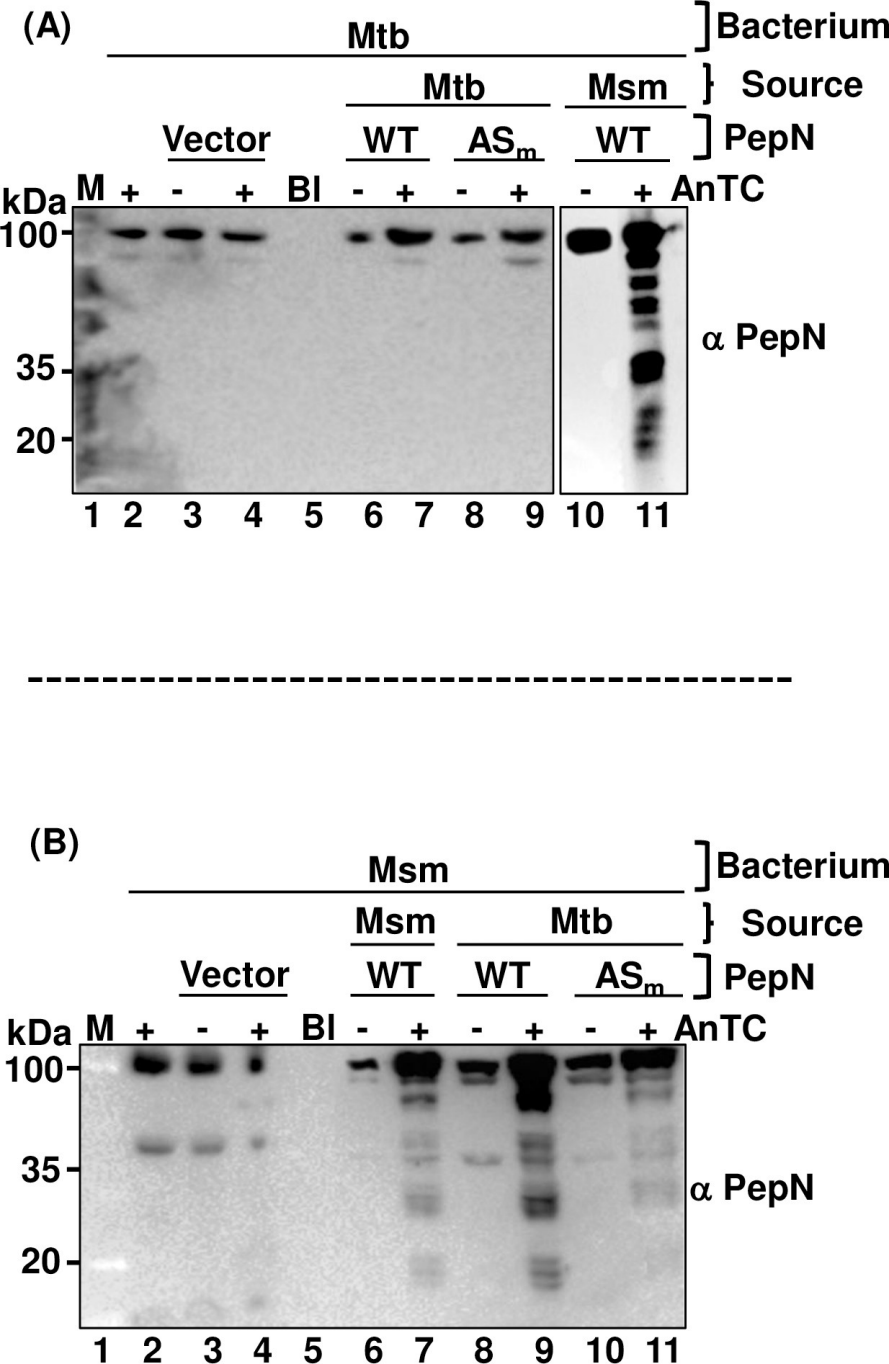
Msm and not Mtb proteolyzes excess of its own PepN. Equal protein lysates from mid-log grown Mtb (A) or Msm (B) and their PepN overexpressing strains were resolved on 10% SDS-PAGE gels, western analysis performed, and steady state levels of PepN monitored. Anti-PepN_Mtb_ antibody was used for detection of PepN. Rabbit polyclonal anti-PepN antibody—(1:2500); anti-Rabbit IgG Goat secondary antibody (1:10000). (A): lane 2—WT Mtb; lane 3 & 4—Mtb with vector alone; lane 6 & 7 –Mtb overexpressing PepN_Mtb_; lane 8 & 9 –Mtb overexpressing mutant PepN_Mtb_; lane 10 & 11 –Mtb overexpressing PepN_Msm_ (B): lane 2—WT Msm; lane 3 & 4—Msm with vector alone; lane 6 & 7 –Msm overexpressing PepN_Msm_; lane 8 & 9 –Mtb overexpressing PepN_Mtb_; lane 10 & 11 –Mtb overexpressing mutant PepN_Mtb_. M- Protein marker.–and + indicate absence or presence of AnTc (100 ng/ml) respectively. Bl–Blank lane. The experiments were independently repeated thrice. ‘PepN’: indicates whether the overexpressing version is wild type or a mutant form (both expressed as episomal). ‘Source’: Indicates the bacterium from where the ‘PepNs’ are derived from. ‘Bacterium’ indicates whether it is Mtb or Msm that expresses those ‘PepNs’. These also express their native PepNs from their genome.

Such opposing dependencies of Mtb and Msm to PepN *in vitro* led us to test if *pepN*_Mtb_ and *pepN*_Msm_ exhibit qualitative/quantitative differences in their transcript and/or steady state levels of PepN. Though *pepN*_Mtb_ and *pepN*_Msm_ exhibited some differences in their transcript levels in rich and minimal media (10.6084/m9.figshare.7873478), overall, by mid-log phase, significant dampening in transcript levels of *pepN* occurred in both Mtb and Msm (10.6084/m9.figshare.7873478). In contrast, western analysis showed both PepN_Mtb_ and PepN_Msm_ steady state levels being uniform across all stages of *in vitro* growth including stationary phase (S6 Fig). Typically, PepN_Mtb_ migrates at ~100 and ~90–95 kDa (Fig 3 and S6B Fig) while PepN_Msm_ migrates at ~ 100, 90–95, 60–63 and 35–40 kDa (Fig 3 and S6A Fig). In both, the 100-kDa form constitutes the major pool.

Uniform steady state levels of both PepNs implies that they either have extended half-lives or possible fine-tuning by the bacteria, for unknown reasons. To test this, we deliberately altered the native steady state PepN_Mtb_ and PepN_Msm_ levels by overexpressing them separately in both WT bacteria. Equal amount of total proteins were loaded (S7A and S7B Fig) and PepN levels evaluated (Fig 3). Mtb well tolerated increased levels of PepN_Mtb_ (Fig 3A—compare lanes 2, 4 and 7; S7C Fig). However, when we expressed *pepN*_Msm_, it proteolyzed over accumulating PepN_Msm_ (Fig 3A—compare lanes 7 and 11). It similarly proteolysed accumulating high levels of 3XFLAG::PepN_Msm_ (S7D Fig). Unlike Mtb, Msm proteolysed excess of its own (PepN_Msm_) (Fig 3B, compare lanes 2, 4 and 7) and foreign PepN (here PepN_Mtb_; Fig 3B, compares lanes 2, 7 and 9). Thus, while Mtb selectively proteolyses over accumulating PepN_Msm_, Msm proteolyses both PepN_Msm_ and PepN_Mtb_. As expected, Mtb and Msm harboring plasmid and vector controls expressed PepN levels similar to their WT controls (Fig 3A and 3B).

We then tested, if WT-Msm discriminates between active and mutant PepN pools. Interestingly, Msm proteolyzes both WT PepN_Mtb_ and mutant PepN_Mtb_ (mPepN_Mtb_; peptidase active site GAMEN mutated to GAAAN and zinc-binding motif HEXXH mutated to AAXXH) with equal efficiency (Fig 3B, compare lanes 9 and 11). We speculate that such proteolytic activity of Msm towards its own and foreign 100 and 95-kDa PepN forms possibly generates the 60–63 and 35–40 kDa minor forms (S6A Fig).

### Msm and not Mtb secretes portion of its excess PepN into spent media

Mass spectrometric (MS) analysis of spent media (SM) of Mtb lab cultures had established earlier that Mtb secretes PepN [4]. Using similar approach, we determined that Msm too secretes PepN (Table 1; see Materials & Methods for details). Though western analyses detected secreted PepNs, they required loading of atleast 15 fold excess culture filtrate proteins (S8 Fig). Therefore, we employed MS approach to detect secreted PepNs.

We had observed that, in Mtb, though *pepN* transcripts significantly scale down by mid-log (10.6084/m9.figshare.7873478), PepN steady state levels remain uniform across all phases of *in vitro* growth (including stationary phase; S6B Fig). As a result, we hypothesized that unlike Msm, Mtb perhaps maintains uniform PepN_Mtb_ levels by secreting out excess PepN. To evaluate this, through MS, we monitored PepN levels in SM of Mtb culture overexpressing its PepN. Contrary to our speculation, Mtb did not secrete any excess PepN_Mtb_ into SM (Table 1). Opposingly, Msm when overexpressing its own PepN, secreted it atleast 3-fold more (see number of peptides; Table 1) suggesting that it indeed maintains steady state levels of PepN_Msm_ by not only proteolyzing but also by secreting excess PepN_Msm_. As a control, we monitored and found EsxB (a well-established secreted protein in mycobacteria) levels uninfluenced by PepN overexpression across all strains evaluated (Table 1).

### PepN_Mtb_ and PepN_Msm_ interactomes are markedly different

**Fig 4:**
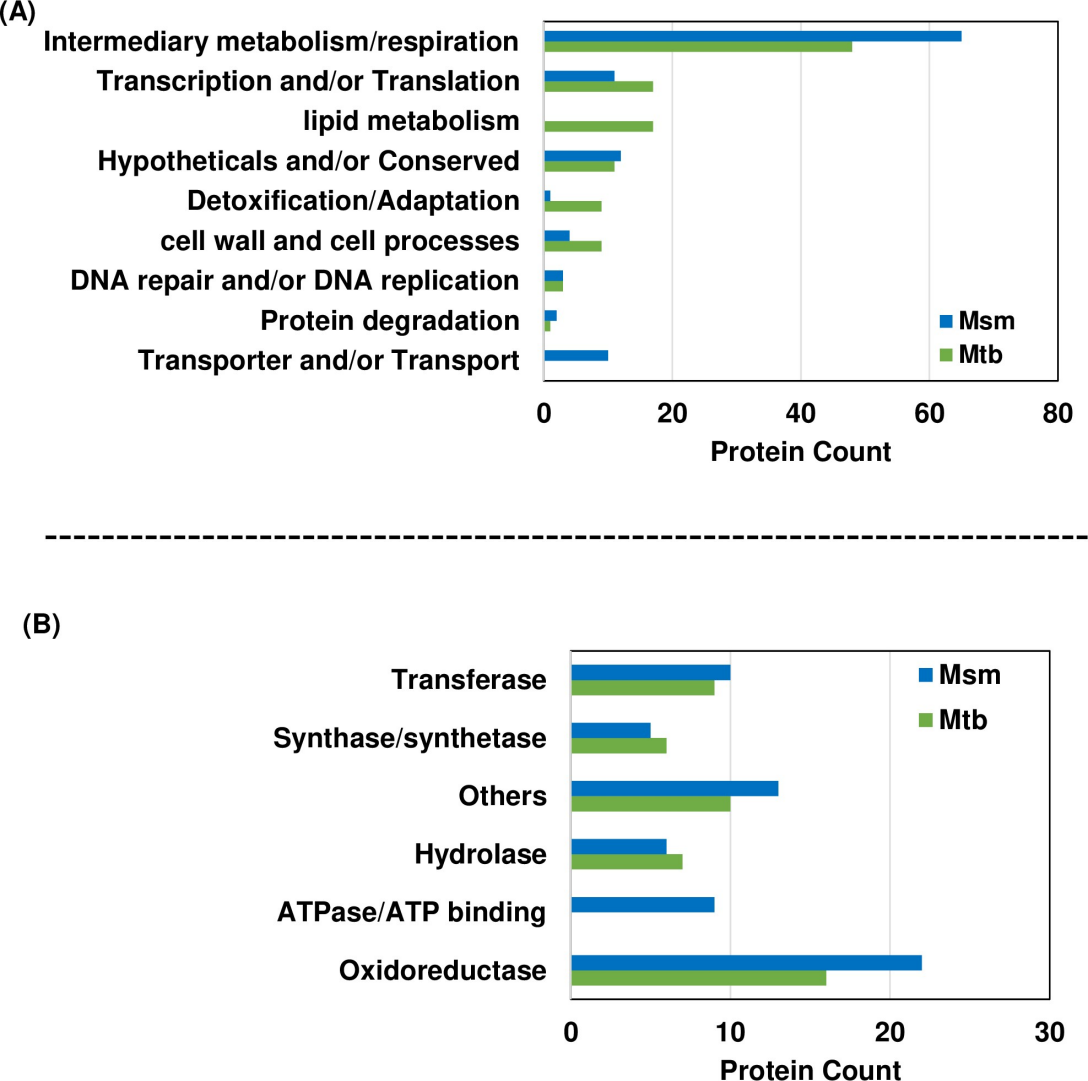
PepN_Mtb_ and PepN_Msm_ interactomes are markedly different. **(A)** Comparative representation of biological processes/functional groups into which the PepN_Mtb_ and PepN_Msm_ interactomes fall. **(B)** Comparative breakdown of intermediary metabolism/respiration group into different subgroups of enzymes to which PepN_Msm_ and PepN_Mtb_ interactomes belong. Detailed grouping shown in S3 and S4 Tables respectively. Biological triplicates were processed for MS/MS.

As an essential aminopeptidase (Fig 2), PepN_Msm_ might interact and modulate several Msm proteins required for Msm growth and survival. To test this, we co-immunoprecipitated PepN_Msm_ together with its interacting partners and identified them by tandem MS. We employed appropriate controls (S9 Fig) and subtracted out non-specific proteins (see Materials and Methods), we identified 107 PepN_Msm_-specific interacting partner proteins (S3 Table). Among them, 60% are enzymes involved in diverse intermediary metabolism and/or respiration activities, the so-called workhorses of cellular growth and survival (Fig 4A and 4B; S3 Table). Thirty percent of these enzymes are oxidoreductases. Another 30% of the enzymes are a combination of synthases, synthetases, transferases & hydrolases. Eighteen percent of the interactome constituted ATPases or ATP-binding proteins (Fig 4B, S3 Table). The remaining potential interactors included (i) ribosomal proteins, translation initiation factors, Ribonuclease E all necessary for survival and (ii) ABC transporters, SecA2, SecY, signal peptidase 1, Trigger factor and Tat pathway signal sequence domain protein all involved in proteins transport across inner membrane. Importantly, PepN_Msm_ also interacted with Pup deamidase/depupylase and proteasomal core subunit beta, both necessary components of protein degradation machinery (S3 Table). Thus, >90% of PepN_Msm_ interactome constitute several important housekeeping proteins that may have significant role in Msm survival and growth *in vitro*.

Since PepN_Mtb_ reaches cytosol (Fig 1) and remains nonessential for *in vitro* growth (S5 Fig), we speculated that it might interact with and may modulate levels of several host and pathogen proteins required *in vivo*. We attempted to co-immunoprecipitate interacting pathogen and host proteins from Mtb-infected THP-1 lysates (see materials and methods). While we could easily enrich for PepN and several host proteins (details beyond the scope of this manuscript), MS was not sensitive enough to directly detect pathogen proteins present in THP-1 environment.

Consequently, we co-immunoprecipitated PepN_Mtb_ and its partners from Mtb lysate, applied essential controls and identified 114 PepN_Mtb_-specific partner proteins (S4 Table). Surprisingly, one third of PepN_Mtb_ interactome constitute secreted proteins of Mtb. Around 55% of its interactome constituted proteins nonessential [22] for *in vitro* growth of Mtb (S4 Table). Only 40% of the total interactome constitute proteins involved in intermediary metabolism/respiration functions (Fig 4A). Of those enzymes though 1/3^rd^ constitute oxidoreductases (Fig 4B), 50% of them are again non-essential for Mtb growth *in vitro*. Similarly, around 50% of interacting hydrolases and transferases are nonessential. Around 15% of PepN_Mtb_ interactome uniquely constitute proteins of lipid metabolism (Fig 4A; S4 Table), 85% of which are again nonessential. Additionally, around 10% of PepN_Mtb_ interactome contain proteins involved in detoxification/adaptation processes (Fig 4A), most of them again nonessential for Mtb growth *in vitro* (S4 Table). Most hypotheticals/conserved proteins with no functions (8 of 10), DNA repair/replication proteins (2 of 3) and cell wall and cell processes–mediating proteins (6 of 9) were again found to be nonessential (Fig 4A; S4 Table). Thus, more than 55% of PepN_Mtb_ interactome constitute proteins that are not essential to Mtb growth *in vitro*. Notably, only eight proteins among the PepN_Mtb_ and PepN_Msm_ interactomes are common (S3 and S4 Tables). However, both interactomes constituted proteins of various functional groups [18] (Fig 4A).

To eliminate any concerns that the final interactome lists do not contain proteins in random, we queried both interactomes for interacting networks (using STRING–an interaction network database) [46]. We then imported these networks into Cytoscape [47], assigned colors to each functional group and manually arranged them (10.6084/m9.figshare.7873484). Around 80–83% of PepN_Mtb_ and PepN_Msm_ interactome proteins fell into these interacting networks (10.6084/m9.figshare.7873484; 10.6084/m9.figshare.7873487), validating that our co-immunoprecipitates are highly relevant. Finally, based on protein abundance (eMPAI values of > or < 0.75), we also grouped the interactome proteins and fetched known interactors (not present in interactome lists) from STRING [46]. Our fetched primary protein interactors and their second neighbors differed between PepN_Mtb_ and PepN_Msm_ again suggesting their functional diversity (10.6084/m9.figshare.7873487).

## Discussion

Our search for potential Mtb effectors among its aminopeptidases pool led us to PepN. Since it is (i) reported to be secreted into SM [4]; (ii) not essential for Mtb’s *in vitro* growth [22], and (iii) essential for Mtb’s growth *in vivo* [24], we speculated that this might be a potential effector in hosts. Our *in vitro* macrophage infection studies with Mtb showed that PepN not only secretes into SM [4] but also reaches macrophages (Fig 1). Presence of (i) a C-terminal ERAP1_C-like domain [19] that resembles ERAP1, an essential host aminopeptidase of ER [39] and (ii) identification of ER-homing like signals along its length (S2 Fig) furthered us to hypothesize that PepN not only enters macrophages but also reaches ER. However, surprisingly, our THP-1 infection experiments showed that PepN barely co-localizes with GRP94, an ER-specific marker. (Fig 1). Further evaluation also indicated that PepN fails to localize with THP-1’s nuclei (Fig 1) and lysosomes (S3 Fig). Thus, our *in vitro* infection experiments with THP-1 indicate that PepN is largely localized to host cell cytosol. Himar 1 insertion into *pepN*_Mtb_ showed that mutated PepN led to attenuation of Mtb-mediated virulence in mice [24]. In contrast, a Tn*5370* insertion into *pepN* facilitated Mtb to become hypervirulent in BALB-C mice [23]. It is unclear at this point as to what probably is the function of PepN_Mtb_ post its delivery into host macrophages.

Thus, if PepN_Mtb_ has evolved to play a major role *in vivo* [23, 24], we wondered what role PepN_Msm_ has in store. Msm not only expresses *pepN*, but also maintain uniform steady state levels across all growth stages *in vitro* (S6 Fig). Our failure to generate an MsmΔ*pepN* indicates PepN’s essential role during Msm growth *in vitro*. While we also confirmed its necessity for *in vitro* growth by fusing a degradation tag, SsrA (Fig 2A and 2B), emergence of one slow-growing revertant after THREE weeks on selection further suggested PepN_Msm_ role towards Msm survival. Uniform levels of PepN_Msm_ indicates possible regulation. Interestingly, failure to even knockdown PepN_Msm_ protein levels by CRISPRi-Cas9 [44] (0.6084/m9.figshare.7873469) reinforces such regulation. Our overexpression studies also indicate that Msm senses excess levels of PepN and proteolyzes it (Fig 3B). Interestingly, Msm proteolyzes both native and foreign PepN with equal efficiency (Fig 3B). It seems to sense quantity over quality as it proteolyzes the active site and zinc-binding motif double mutant, mPepN_Mtb_ with similar efficiency (Fig 3B).

One wonders why proteolysis instead of transcriptional or post-translational modifications is the way to fine-tune protein level of an aminopeptidase. Most M1 members play pivotal roles in survival, cell maintenance, growth and development, virulence and pathogenesis [11]. They normally cleave proteins at their lysines, alanines, arginines and leucines [18]. Our co-immunoprecipitation studies showed more than 90% of PepN_Msm_ partners to be housekeeping proteins (S3 Table). Perhaps managing their cellular abundance requires active role of several proteases/peptidases including PepN. Thus, expressing altered PepN levels might have global consequences including death and hence proteolysis of excess PepN becomes a necessary step. We carved out gel pieces at the 60–70 and 30–50 kDa positions, performed MS/MS and detected peptides unique to PepN_Msm_ minor forms (60–63 and 35–40 kDa) confirming that those signals in western are indeed specific to PepN. To also ensure that no partially proteolysed (the 60–63 and 35–40 kDa forms) PepN remain active in the cell, perhaps Msm secretes the excess PepN out too (Table 1). Interestingly, MS of both 60–63 and 35–40 kDa forms detected peptides that span the entire length of PepN_Msm_ indicating that they are indeed proteolytic products of full length PepN_Msm_ and not its truncated forms.

In contrast to Msm, Mtb tolerates its own PepN and selectively proteolysis the non-native kind (Fig 3A). Additionally, it does not secrete out any excess PepN it continues to tolerate (Table 1). If PepN_Mtb_ were to have a specific *in vivo* role in host cytosol, this control of secretion makes sense, as it needs to modulate/proteolyse host and pathogen protein levels only to the extent necessary. Interestingly, when we monitored PepN_Mtb_ localization especially of THP-1 infected with Δ*pepN* expressing PepN_Mtb_ (in trans), despite higher levels of induction (~96 h; S7C Fig), we did not find excess PepN_Mtb_ signals (fluorescence intensity). Its structure resemblance to ERAP1 (10.6084/m9.figshare.7873451) and localization to cytosol (Fig 1) lets us speculate that it modulates members of its pathogen interactome that reach the host. Almost one third of PepN_Mtb_ interacting partners are Mtb secreted proteins. Given the aminopeptidase role, and it ability to modulate virulence [23,24], it is tempting to hypothesize that PepN_Mtb_ may cleave pathogen and host proteins in host macrophages to regulate virulence levels. We are currently performing experiments to evaluate these possibilities.

Thus, our comparative analyses provides ample insights into common and possible opposing roles for PepN_Mtb_ and PepN_Msm_. Their divergent interactomes (S3 and S4 Tables) to 78% identity (10.6084/m9.figshare.7873274) sheds light on their potential specific roles. Only eight proteins were common to both interactomes (S3 and S4 Tables). Though the remaining 106 Mtb and 99 Msm proteins fell into common functional groups (Fig 4A and 4B), proteins constituting each category were strikingly different (Fig 4; S3 and S4 Tables). For eg. one third of PepN_Mtb_ interactome contained secreted proteins potentially involved in lipid metabolism, adaptation to stress, detoxification, and intermediary metabolism, all essential for pathogen’s survival in the host. Unlike PepN_Mtb_ interactome, PepN_Msm_ interactome lacked any proteins involved in lipid metabolism (S3 and S4 Tables).

In contrast, one fifth of the PepN_Msm_ interactome consist of oxidoreductases that help in redox maintenance (S3 Table). Around 10% of the PepN_Msm_ interactome consists of ABC transporters or proteins including SecA2, SecY, signal peptidase and Tat pathway signal sequence domain protein all necessary for protein translocation across inner membrane (S3 Table). While SecA2 might play an accessory role in protein translocation, cell cannot survive without SecY [48]. Perhaps PepN_Msm_ plays a role in controlling SecY levels. Additionally, around 15% of the interactome constitutes proteins involved in DNA repair/transcription/translation/protein degradation including DNA polymerase III γ-subunit, DNA partitioning proteins, ribosomal proteins, transcription factors and pup deamidase/depupylase (S3 Table) all again perhaps performing essential functions in Msm. The PepN_Msm_ co-precipitant also included a transglycosylase that has important functions to play in cell wall integrity [49]. Interestingly, PepN_Msm_ interacts with trigger factor (tig) that interacts with several important players including SecA1, a major player of protein translocation [50] (S3 Table and 10.6084/m9.figshare.7873484; 10.6084/m9.figshare.7873487). Tig is a chaperone that binds to nascent proteins to keep them in a translocation compatible confirmation [51]. Such interactions again reinforce the necessity of PepN_Msm_ for *in vitro* growth.

Though, our co-immunoprecipitation studies shed light on possible role of these PepNs, identifying their cognate substrate proteins, evaluating when and how their levels get modulated might be necessary to determine the exact functions of PepN-like aminopeptidases. While this requires the comparative quantitative proteomic analysis between WT Mtb and Msm with their cognate Δ*pepN*s, proteolysis by ATP-independent aminopeptidases are generally considered distal to ATP-dependent steps of proteolysis [52]. This could mean that the potential PepN substrates in both Mtb and Msm backgrounds could be first proteolyzed by other ATP utilizing proteases into peptides before they are handed over to PepNs for further proteolysis. Since PepN_Msm_ is necessary for *in vitro* growth, MsmΔ*pepN* could not be generated and hence the comparative quantitative proteomics could not be performed in Msm. Given the PepN_Mtb_ redundancy (in Mtb growth *in vitro*), we are currently exploring use of quantitative proteomics to determine what host proteins does WT Mtb and MtbΔ*pepN* influence. Perhaps this would shed light on understanding how the pathogen modulates host cellular environment. In summary, we show that despite high homology, the PepN aminopeptidases of the slow-growing pathogenic and fast-growing non-pathogenic mycobacteria have evolved to carry divergent traits that define their host and/or pathogen-specific functions. To the best of our knowledge, there is no report thus far reporting on such divergent traits in any bacterial aminopeptidase.

## Supporting information

S1 Table List of plasmids used in this study. (PDF)

S2 Table List of primers used in this study. (PDF)

S3 Table PepN_Msm_ interactome list. Gene names: Consists of Msm’s gene numbers as MSMEG_XXXX and/or gene names to all UniProt [37] IDs obtained from MS analysis of PepN_Msm_ interactome. GO (Gene Ontology)–Molecular function: Potential biological process/function (as listed in UniProt, [37] to genes listed in column one. Functional Group: Genes in column one were searched in Mycobrowser [18] to identify the major functional group to which they belong. All genes belonging to intermediary metabolism and/or respiration are further sub-grouped in different categories of enzymes.
(XLSX)

S4 Table PepN_Mtb_ interactome list. Gene names: Consists of Mtb H37Rv’s gene numbers as RvXXXX and/or gene names to all UniProt [37] IDs obtained from MS analysis of PepN_Mtb_ interactome. GO (Gene Ontology)–Molecular function: Potential biological process/function as listed in UniProt [37] to genes listed in column one. Secretion: Yes–Proteins corresponding to genes in column one that are secreted into spent media; No—Proteins corresponding to genes in column one that are NOT secreted into spent media; Essentiality: Primarily corresponds to whether the genes in column 1 are essential (Yes) or not essential (No) for Mtb growth *in vitro*; when indicated (B) (next to Yes)—essential both *in vitro* and *in vivo*. Functional Group: Genes in column one were searched in Mycobrowser [18] to identify the major functional group to which they belong. All genes belonging to intermediary metabolism and/or respiration are further sub-grouped in different categories of enzymes.
(XLSX)

S1 Fig PepNs of pathogenic and non-pathogenic mycobacteria structurally differ in their C-terminal ERAP1_C-like domain. Using Expresso [32], a T-Coffee flavor that aligns multiple sequences based on structural information, PepNs from both, the slow-growing pathogenic mycobacteria (Mtb complex) viz. Mtb, *M*. *bovis*, *M*. *caprae*, *M*. *africanum*, *M*. *canetti* and *M*. *leprae* (top left) and fast growing, non-pathogenic mycobacteria representative, Msm were aligned. Expresso identified 3q7j (https://www.rcsb.org/structure/3q7j) as the reference structure, to which it aligned PepNs of Mtb complex and PepN_Msm_. Numbers to right of mycobacteria indicate structure-based sequence identity scores. Numbers to either end of each sequence denotes its cognate first and the last amino acid in each block. Transparent cyan box: N-terminal M1 peptidase domain; Transparent red box: ERAP1_C domain; Transparent green boxes—M1 peptidase active site (GAMEN) and zinc-binding motif (HEMAH); structure-based sequence alignment color key: Good–pink; average–yellow; poor–green; In the consensus line: cons–consensus; _*_—identical amino acids;. – strongly similar amino acids; .–weakly similar amino acids; () (blank space)–different amino acids.
(TIF)

S2 Fig PepN_Mtb_ harbors potential ER-homing like signals. When PepN_Mtb_ is used as query sequence, LocSigDB [42] identified potential ER-homing signals (indicated in small rectangular boxes with thin blue lines, each box has three amino acids). Large transparent green and red box: M1 peptidase domain (Green—Peptidase M1 N-terminal domain; Red—Peptidase family M1 domain) and large transparent purple box: C-terminal ERAP1_C domain. The consensus signal sequences identified are at the bottom left, the co-ordinates of the identified amino acids are in the middle and the predicted localization of the query protein is to the bottom right.
(TIF)

S3 Fig Both PepN_Mtb_ and PepN_Msm_ do not localize to lysosomes. Immunofluorescence-based localization analysis (see materials and methods for protocol) of PepN_Mtb_ in THP-1 infected with either WT Mtb (top two panel rows); MtbΔ*pepN* (middle (third) panel row) or MtbΔ*pepN* expressing *pepN*_Msm_ (bottom two panel rows). **Panel columns:** First: DAPI to track nuclei (blue); Second: virulent Mtb expressing mCherry (pink)—to locate infected THP-1; Third: Lysotracker green (green); Fourth: PepN (red); Final: merger of first four panels (of each row); Scale: 10 μm. Atleast 300 infected macrophages were visualized for confirming consistency of observed results. The immunofluorescence data is a representation of three independent experiments. Each independent experiment had biological duplicates.
(TIF)

S4 Fig PepN_Msm_ harbors potential ER-homing like signals. When PepN_Msm_ is used as query sequence, LocSigDB [42] identified potential ER-homing signals (indicated in small rectangular boxes with thin blue lines, each box has three amino acids). Large transparent green and red box: M1 peptidase domain (Green—Peptidase M1 N-terminal domain; Red—Peptidase family M1 domain) and large transparent purple box: C-terminal ERAP1_C domain. The consensus signal sequences identified are at the bottom left, the co-ordinates of the identified amino acids are in the middle and the predicted localization of the query protein is to the bottom right. Unlike PepN_Mtb_, PepN_Msm_ lacks any [HK]x{1}K -like sequence.
(TIF)

S5 Fig MtbΔ*pepN* generation and its confirmation. Homologous recombination-based strategy with SacB as counter selection marker [45] was adapted to generate MtbΔ*pepN*. **(A)** indicates schematics of the strategy adapted. **Step 1:** Rectangular dotted (dull grey) boxes indicate PCR-amplified upstream and downstream regions of *pepN* that were cloned into the suicidal vector pKA1 to obtain pNS22. ‘X’ drawing indicates potential recombination occurring regions between *pepN*_Mtb_ (in the genome) and regions of pNS22. P1 to P9 –primers used for confirmation/analyses. Single line arrows below/above primers indicate forward and reverse directions. Broad arrows in pNS22 and genome indicate open reading frames. **Step 2:** schematics of single crossover that occurred between the upstream region to *pepN*_Mtb_ and pNS22 upstream fragment. Upon single crossover, the entire pNS22 is recombined into the *pepN*_Mtb_ locus. **Step 3:** schematics of *pepN*_Mtb_ deletion that occurred as a result of the second recombination between downstream region to *pepN*_Mtb_ and pNS22 downstream fragment. This led to *pepN*_Mtb_ being replaced by the Hygromycin resistance cassette flanked by unidirectional *loxP* sites. **Step 4:** schematics of the *pepN*_Mtb_ locus lacking both *pepN*_Mtb_ and Hygromycin resistance cassette. The resistance cassette was removed by use of pCre-Zeo encoded Cre-recombinase. **(B) & (C):** PCR-based validation of Step 2 (of (**a**)); While (**B)** indicates only recombination of pNS22 into the genome, (**C)** indicates recombination of pNS22 in to the *pepN*_Mtb_ upstream region. Three putative Hyg+ single crossover colonies that did not grow on 10% sucrose were selected for PCR-based single crossover screening at the upstream region (lanes 2–4) with primers P3 and P4 (**B**) and P4 and P5 (**C**). In (**B):** P3 and P4 primers amplify a 1.1 kb region from both the single crossovers colonies and from pNS22 (+ve: lane 5). As expected, the primers did not amplify any 1.1 kb fragment from the WT Mtb genomic DNA (lane 6). In **(C):** P4 and P5 primers amplify a 1.3 kb region from only the single crossovers colonies (at their upstream region). Neither pNS22 (-ve: lane 5) nor genomic DNA of WT Mtb (lane 6) show the 1.3 kb amplicon indicating that the single crossover occurred at the upstream region to *pepN*_Mtb_ as designed. M—1 kb ladder (lane 1 in (**C**) and (**C**)) with specific sizes indicated to the left. **(D):** PCR-based validation of Step 3 (of (**A**)); While the **left section** (to broken line on image) indicates double crossover into the *pepN*_Mtb_ downstream region, the **right section** (to broken line on image) indicates double cross over alone. Three putative Hyg+ double crossover (putative Δ*pepN*) colonies that grew on 10% sucrose were selected for PCR-based double crossover screening at the downstream region (lanes 1–3) with primers P6 and P8 (left section) and P6 and P7 (right section). In **left section:** P6 and P8 primers amplify a 1.4 kb region from putative Δ*pepN* colonies only (at their downstream region). Neither pNS22 (-ve: lane 4) nor genomic DNA of WT Mtb (lane 5) show the 1.4 kb amplicon indicating that the double crossover occurred at the downstream region to *pepN*_Mtb_ as designed. In **right section:** P6 and P7 primers amplify a 1.1 kb region from the double crossover (putative Δ*pepN*) colonies (lanes 6–8) and from pNS22 (+ve: lane 9). As expected, the primers did not amplify any 1.1 kb fragment from the WT Mtb genomic DNA (lane 10). M—1 kb ladder (lane 11) with specific sizes indicated to the left. **(E):** PCR-based validation of Step 4 (of (**A**)): Two Δ*pepN* colonies with accurate double crossover as verified in (b), (c) and (d) are PCR-amplified with P2 and P9 primers (left of M—a 1kb ladder) and yield a ~ 0.47 kb amplicon indicating deletion of *pepN*_Mtb_ and removal of Hyg + cassette. In contrast, the WT Mtb genomic DNA (WT lane) yielded ~ 2.7 kb amplicon indicating presence of *pepN*_Mtb_. When P1 and P7 primers were used (right of M lane), the WT genomic DNA yield a 1.3 kb amplicon indicating presence of *pepN*_Mtb_. The two Δ*pepN* colonies did not yield any amplicon as P1 lies internal to *pepN*_Mtb_ confirming the colonies as deleted of *pepN*_Mtb_. Go Taq polymerase was used to perform all PCRs (B-E). **(F):** Confirmation of Δ*pepN*_Mtb_ by western analysis. Mid-log grown cultures of Mtb (H37Ra, H37Rv and the two Δ*pepN*_Mtb_ colonies (from (e)) were pelleted down, lysed by bead beating, total proteins boiled in 1X Laemmli’s buffer and loaded on a 10% SDS-PAGE. 10 fold more total protein was loaded in the two Δ*pepN*_Mtb_ colonies lanes. Total protein of *E*. *coli* C41(DE3) harboring pNS23 (S1 Table; expressing *pepN*_Mtb_) was used as positive control. Mid-log culture of Msm (mc^2^155) were pelleted down, lysed by bead beating, total proteins boiled in 1X Laemmli’s buffer and loaded on a 10% SDS-PAGE to detect for the major and minor forms of PepN_Msm_ that migrate in the gel. The nitrocellulose membrane with transferred proteins was developed with Anti-PepN (1:2500) as primary and anti-rabbit Goat IgG as secondary (1:10000). M—protein marker. **(G):** Confirmation of Δ*pepN*_Mtb_ by Southern analysis. Overnight digested (separately with *Pvu*I and *Not*I) genomic DNA (4 ug) of WT Mtb (lane 1 & 2 respectively) and MtbΔ*pepN* (lane 4 and 5) were resolved electrophoretically on 0.8% agarose gel. Lane 3 contains 1 kb Plus Ladder (M); Lane 8 & 9–10 & 20 ng DIG labelled probe. After resolving, the gel was processed as per protocol (see materials and methods) and probed with DIG-labelled amplicon of 421 bp (amplified with KAP8 and KAP474 as indicated in (**A**)) to detect specific size fragments (as mentioned to the right of the Hybond Nylon + membrane image). The signal in lanes 8 and 9 indicate the correct size of the probe amplicon. A standard measuring ruler (right of blot) was used to measure the distance the marker fragments (unlabeled) had resolved for accurate estimation of signal mobility. The signal was obtained by using CSPD substrate and chemiluminescent signal generated was monitored and recorded on the gel documentation system (BioRad, USA). The probe location is as depicted.
(TIF)

S6 Fig Steady state levels of Mtb and Msm PepNs are uniform across all *in vitro* growth phases. To compare steady state levels of Msm (A; mc^2^155) and Mtb (B; H37Rv) PepNs across different *in vitro* growth phases (as shown in A and B), ~250 ml cultures each were first grown in rich—(7H9 + ADC/OADC) and minimal media (Sauton’s). Then, aliquots were sampled (20 ml each), washed, lysed (by bead beating) and equal total protein (estimated by BCA kit (Thermo Fisher Scientific, USA)) resolved on 10% SDS-PAGE gels and westerns performed. Anti-PepN antibody (1:2500) was used for detecting PepN. Anti-Rabbit IgG Goat secondary antibody—1:10000; M- Protein marker. The western blots represent three independent experiments, each with biological duplicates. Optical density (A_600nm_) were recorded at different time-points as indicated. The data for each time point is thus mean ± SE.
(TIF)

S7 Fig Msm, unlike Mtb, proteolyzes its excess PepN. To normalize equal protein amounts (estimated by BCA kit (Thermo Fisher Scientific, USA)) for western analyses (Fig 3), total proteins of lysates of mid-log grown Mtb (A) or Msm (B) and their PepN overexpressing strains (A and B) were resolved in 10% SDS-PAGE gels and coomassie stained. **(A):** Lane 2—WT Mtb; Lanes 3 & 4—Mtb with vector; Lanes 5 & 6 –Mtb overexpressing PepN_Mtb_; Lanes 7 & 8—Mtb overexpressing mutant PepN_Mtb_; and Lanes 9 & 10 –Mtb overexpressing PepN_Msm_. **(B):** Lane 2—WT Msm; Lanes 3 & 4—Msm with vector; Lanes 5 & 6 –Msm overexpressing PepN_Msm_; Lanes 7 & 8 –Msm overexpressing PepN_Mtb_; and (iv) Lanes 9 & 10 –Msm overexpressing mPepN_Mtb_ **(C):** **Mtb tolerates over accumulation of its PepN even after 96 h of induction**. Equal protein amounts from lysates of mid-log grown Mtb overexpressing PepN_Mtb_ (induced for 48 and 96 h) were resolved in 10% SDS-PAGE gels, western analysis performed and accumulating levels of PepN_Mtb_ monitored. Anti-PepN_Mtb_ antibody (1:2500) was used for detecting PepN. Anti-Rabbit IgG Goat secondary antibody—1:10000; M- Protein marker. **(D):** **Mtb selectively proteolyzes excess 3XFLAG::PepN**_**Msm**_. Equal protein amounts from lysates of mid-log grown Mtb overexpressing either 3XFLAG::PepN_Msm_ (blot to the left) or 3XFLAG::PepN_Mtb_ (blot to the right) were loaded onto 10% SDS-PAGE gels, western analysis performed and accumulating levels of PepN_Mtb_ and PepN_Msm_ monitored with FLAG antibody (Sigma Aldrich, USA). M—Protein marker.–and + indicate absence or presence of AnTc (100 ng/ml) respectively. Rabbit polyclonal anti-PepN antibody—(1: 2500) and anti-Rabbit IgG Goat secondary antibody (1:10000). Mouse FLAG specific monoclonal antibody–(1: 5000).
(TIF)

S8 Fig PepN_Mtb_ and PepN_Msm_ get secreted into SM of lab cultures. For validating secretion of PepN_Mtb_ and PepN_Msm_ as evaluated by MS/MS (Table 1), exponential phase lab cultures of WT Msm (mc^2^155) and Mtb (H37Rv) were grown, cell pellets collected, lysed by beat beating, lysate filtered twice and total proteins estimated by BCA kit (Thermo Fisher Scientific, USA). The spent media were also filtered twice, TCA precipitated and total protein estimated. Equal amount of lysates (left blots) and 10-fold higher amount of culture filtrate (CF) proteins (right blots–CF) were loaded onto 10% SDS-PAGE, proteins resolved and westerns performed. Blots were developed with specific anti-PepN antibody (α PepN blots) and Hsp65-specific antibody (Abcam, UK). Hsp65 (Rv0440 in Mtb and MSMEG_0880 in Msm) is used as lysis control. Anti-PepN_Mtb_ antibody (1:2500) and anti-Rabbit IgG Goat secondary antibody (1:10000) were used for detection of PepN. Anti-Hsp65 antibody (1:2500) and anti-mouse IgG Goat secondary antibody (1:10000) were used for detection of Hsp65. White tiny bands in each blot indicate protein markers (kDa). These are representative blots of three independent experiments and their biological duplicates.
(TIF)

S9 Fig PepN_Mtb_ and PepN_Msm_ interactomes are markedly different. **(A):** **Light blue oval:** combined co-immunoprecipitant protein numbers of immunoprecipitated with beads alone; PI_Mtb_—WT Mtb lysate co-immunoprecipitated with pre-immune sera; KOAb—*pepN*_Mtb_ KO lysate co-immunoprecipitated with plus anti-PepN antibodies. **Light yellow oval:** WT_Mtb_Ab—co-immunoprecipitant protein numbers obtained by co-immunoprecipitating WT Mtb lysate with anti-PepN antibodies; **Light green and pink ovals:** KOC_wt_Ab and KOC_m_Ab (respectively)—co-immunoprecipitant protein numbers obtained by co-immunoprecipitating lysates of *pepN*_Mtb_ KO complimented with either *pepN*_Mtb_ or m*pepN*_Mtb_ respectively, both co-immunoprecipitated with anti-PepN antibodies. **Red transparent oval:** indicate PepN_Mtb_ interactome protein numbers (115; S4 Table) that are found common to (i) WT_Mtb_Ab & KOC_m_Ab (20 proteins); (ii) KOC_wt_Ab and KOC_m_Ab (31 proteins); (iii) WT_Mtb_Ab & KOC_wt_Ab & KOC_m_Ab (36 proteins) and (iv) unique to KOC_m_Ab (28 proteins; Fig 4A; see discussion). **(B):** **Blue circle:** WT_Msm_Ab—co-immunoprecipitant protein numbers obtained by co-immunoprecipitating WT Msm lysate with beads and anti-PepN antibodies; **Yellow circle:** combined co-immunoprecipitant protein numbers of NoAb_Msm_ + PI_Msm_; NoAb_Msm_—WT Msm lysate co-immunoprecipitated with beads alone; PI_Msm_—WT Msm lysate co-immunoprecipitated with beads and pre-immune sera. **(A and B):** Protein lists from each group were fed into Venny (http://bioinfogp.cnb.csic.es/tools/venny/index2.0.2.html), venn diagrams generated and common and unique proteins identified.
(TIF)
