# Supplementary material for: Comparative analysis of homologous aminopeptidase PepN from pathogenic and non-pathogenic mycobacteria reveals divergent traits
Source: PLoS One. 2019 Apr 10;14(4):e0215123. doi: 10.1371/journal.pone.0215123 (PMC6457555; doi:10.1371/journal.pone.0215123)
Supplement: S1 Table — (PDF) [file pone.0215123.s001.pdf]

S1 Table. List of plasmids used in this study.

Sl. No.	CONSTRUCT	PLASMID NAME	REFERENCE
1	upstream and downstream sequence to *pepN*$_{Mtb}$ in pKA1	pNS22	This Study
2	*pepN*$_{Mtb}$ in pET28a	pNS23	This Study
3	*pepN*$_{Mtb}$ in pDONR221	pNS24	This Study
4	*pepN*$_{Mtb}$ in pTetSG	pNS25	This Study
5	3X *flag:: pepN*$_{Msmeg}$ in pDONR221	pNS26	This Study
6	3X *flag:: pepN*$_{Msmeg}$ in pTetSG	pNS27	This Study
7	*pepN*$_{Msmeg}$ in pDONR221	pNS28	This Study
8	*pepN*$_{Msmeg}$ in pTetSG	pNS29	This Study
9	3X *flag::pepN*$_{Mtb}$-GAMEN into pDONR221	pNS30	This Study
10	3X *flag::pepN*$_{Mtb}$-GAMEN-HEXXH into pDONR221	pNS32	This Study
11	3X *flag::pepN*$_{Mtb}$-GAMEN-HEXXH into pTetSG	pNS35	This Study
12	Last 1 kb of *pepN*$_{Msmeg}$*::ssrA* into pKA1	pNS36	This Study
13	Last 1 kb of *pepN*$_{Msmeg}$*::ssrA* into pKA2	pNS38	This Study
14	Last 1 kb of *pepN*$_{Msmeg}$ into pKA2	pNS39	This Study
15	Gateway entry vector	pDONR221	Invitrogen
16	Tet-inducible Gateway destination vector for mycobacteria	pTetSG	[1]
17	*E. coli* expression vector	pET28a	Novagen
18	pJM1 derivative with extended MCS (Hyg^R)	pKA1	This study
19	pKA1 with Kanamycin as selection marker	pKA2	This Study

Reference

1. Garces A, Atmakuri K, Chase MR, Woodworth JS, Krastins B, Rothchild AC, et al. EspA Acts as a Critical Mediator of ESX1-Dependent Virulence in Mycobacterium tuberculosis by Affecting Bacterial Cell Wall Integrity. PLoS Pathogens. 2010;6. doi:10.1371/journal.ppat.1000957
