# Supplementary material for: Comparative analysis of homologous aminopeptidase PepN from pathogenic and non-pathogenic mycobacteria reveals divergent traits
Source: PLoS One. 2019 Apr 10;14(4):e0215123. doi: 10.1371/journal.pone.0215123 (PMC6457555; doi:10.1371/journal.pone.0215123)
Supplement: S2 Table — (PDF) [file pone.0215123.s002.pdf]

S2 Table. List of primers used in this study.

Sl. #	PRIMER	SEQUENCE	REMARKS
1	KAP5	GGGGACAAGTTTGTACAAAAAAGCAGGCTAGTAAGAAGGAGATATACATATG	F: *pepN*$_{Mtb}$-2nd-rd Gateway
2	KAP6	GGGGACCACTTTGTACAAGAAAGCTGGGTC	R: *pepN*$_{Mtb}$-2nd-rd Gateway
3	KAP8	GAAGGAGATATACATATGGTGGCCCTTCCAAACC	F: *pepN*$_{Mtb}$-1st-rd Gateway and F:probe
4	KAP11	GTACAAGAAAGCTGGGTCTTACTAGCCGTCAGCGTCGAAGTTGCG	R: *pepN*$_{Mtb}$-1st-rd Gateway
5	KAP225	CAACGCCGCCGCAGCCGAAAACGCCGGCGCGGTGACCTTCTTGGAGGACTACGTCTTCCG	F: *pepN*$_{Mtb}$-GAMEN mutation
6	KAP226	GGCGTTTTCGGCTGCGGCGGCGTTGAATTCGGGGACGAAGAGCTGGTCGTACTTGCCGAACGCGTATGG	R: *pepN*$_{Mtb}$-GAMEN mutation
7	KAP227	GTGCTGGCCGCCATGGCCCACATGTGGTTCGGCGACCTGGTCACCATGACCTGGTGGGAC	F: *pepN*$_{Mtb}$-HEXXH mutation
8	KAP228	GTGGGCCATGGCGGCCAGCACGGTCTCCGCGCGCCGCTCATAGGATGCCCGGGTGACCTTGCTG	R: *pepN*$_{Mtb}$-HEXXH mutation
9	KAP307	AGTGTGGATATCAGCTTGCCGGCTTCCTCGCCGCGCGGAATC	F: Upstream to *pepN*$_{Mtb}$
10	KAP308	TGACAGACTAGTATTGGAGTAGTGGCAGTCGGCGTCGACG	R: Upstream to *pepN*$_{Mtb}$
11	KAP309	TGACGACTCGAGCGCAACTTCGACGCTGACGGCTAGCCCTC	F: Downstream to *pepN*$_{Mtb}$
12	KAP315	AGCTCATATGGTGGCCCTTCCAAACCTCACGCGGGAC	F: *pepN*$_{Mtb}$-pET28a
13	KAP316	TCTAGAGCTCCTAGCCGTCAGCGTCGAAGTTGC	R: *pepN*$_{Mtb}$-pET28a
14	KAP317	GAAGGAGATATACATATGGTGGCACTTCCCAACCTCACCCGTGATCAG	F: *pepN*$_{Msmeg}$-1st-rd Gateway
15	KAP319	GTACAAGAAAGCTGGGTCTTAGCTCACGTCGAACGCGCGGGCACGCAGGGC	R: *pepN*$_{Msmeg}$-1st-rd Gateway
16	KAP336	AGACATGCATGCTTGGCTGGGCACTCCGCCGGGCGCCGCTG	R: Downstream to *pepN*$_{Mtb}$
17	KAP411	CACCTACCTGGTGGCATTGATCG	F: *pepN*$_{Msmeg}$ RT PCR

18	KAP412	GTACTTGCCGAACGCGTACG	R: *pepN*$_{Msmeg}$ RT PCR
19	KAP413	ACGGACACTTACATCGACGACC	F: *pepN*$_{Mtb}$ RT PCR
20	KAP414	TCCTCCAAGAAGGTCACCGC	R: *pepN*$_{Mtb}$ RT PCR
21	KAP 447	GATCATGGCGCGCCTTAATTAAAGACCCGCGTG CACCGGCTGGCCGTCGGCATCTAC	F: *pepN*$_{Msmeg}$ (3'-1 kb)::*ssrA*
22	KAP463	CCATCCCGAAAAGGAAGACC	F: *SigA*$_{Mtb}$ RT PCR
23	KAP464	TCGAGGTCTGGTTCAGCGTC	R: *SigA*$_{Mtb}$ RT PCR
24	KAP465	TTGAGGTGACCGACGATCTCG	F: *MysA*$_{Msmeg}$ RT PCR
25	KAP466	ACGCCTTGTCCTTCTCGGAC	R: *MysA*$_{Msmeg}$ RT PCR
22	KAP 469	GGCGGCCAGGGCGTAGTCGCGCTGGTTCGAGT CGGCGGCGCTCACGTCGAACGCGCGGGCACG CAGGGCC CGCTCGACGCCCGCGCGGCCT	R: *pepN*$_{Msmeg}$ (3'-1kb)::*ssrA*-1st rd
23	KAP 470	AGCCATATTTAAATAGATCTGCTAGCGGCCGCA CTAGTGGCGGCCAGGGCGTAGTCGCGCTGGTT CGAGTCGGCGGC	R: *pepN*$_{Msmeg}$ (3'-term 1 kb)::*ssrA*-2nd rd
24	KAP 474	CGTCGGCGGTTTCGAATTGCGAGTACAG	R:probe
25	P1	GGAAAAGGCCTCGGGCCGCGACC	F: *pepN*$_{Mtb}$-clean KO screen
26	P2	AGCCATGCGGCCGCACTAGTGGCGGCCAGGGC GTAGTCGCGCTGGTGCGAGTCGGCGGCGCCGT CAGCGTCGAAGTTGCGGGCCCGCAACGATCGC TGCACCGCGG	R: *pepN*$_{Mtb}$-clean KO screen
27	P3	AGTGTGGATATCAGCTTGCCGGCTTCCTCGCCG CGCGGAATC	F: *pepN*$_{Mtb}$-Upstream crossover screen
28	P4	GTGTCACAGCGGACCTCTATTCACAGGGTACGG	R: *pepN*$_{Mtb}$-Upstream crossover screen
29	P5	GGCGTGGTCGGCCCCCAGGTAGACG	F: *pepN*$_{Mtb}$-Upstream crossover screen (Outside)

30	P6	GCGATTCAGGTTCATCATGCCGTCTGTGATGGC	F: *pepN*$_{Mtb}$-Downstream crossover screen
31	P7	AGACATCTCGAGTTGGCTGGGCACTCCGCCGGGCGCCGCTG	R: *pepN*$_{Mtb}$-Downstream crossover screen
32	P8	GCAAGGCCGACGTGGTGCACGAAGACC	R: *pepN*$_{Mtb}$-Downstream crossover screen (Outside)
33	P9	GAAGGAGATATACATATGTCGGGTCTGAACGATATCTTCGAAGCTCAGAAAATCGAATGGCACGAAGGTTCCGTGGCCCTTCCAAACCTCACG	F: *pepN*$_{Mtb}$-clean KO screen

2
