# Supplementary figures and images for: Comparative analysis of homologous aminopeptidase PepN from pathogenic and non-pathogenic mycobacteria reveals divergent traits
Source: PLoS One. 2019 Apr 10;14(4):e0215123. doi: 10.1371/journal.pone.0215123 (PMC6457555; doi:10.1371/journal.pone.0215123)

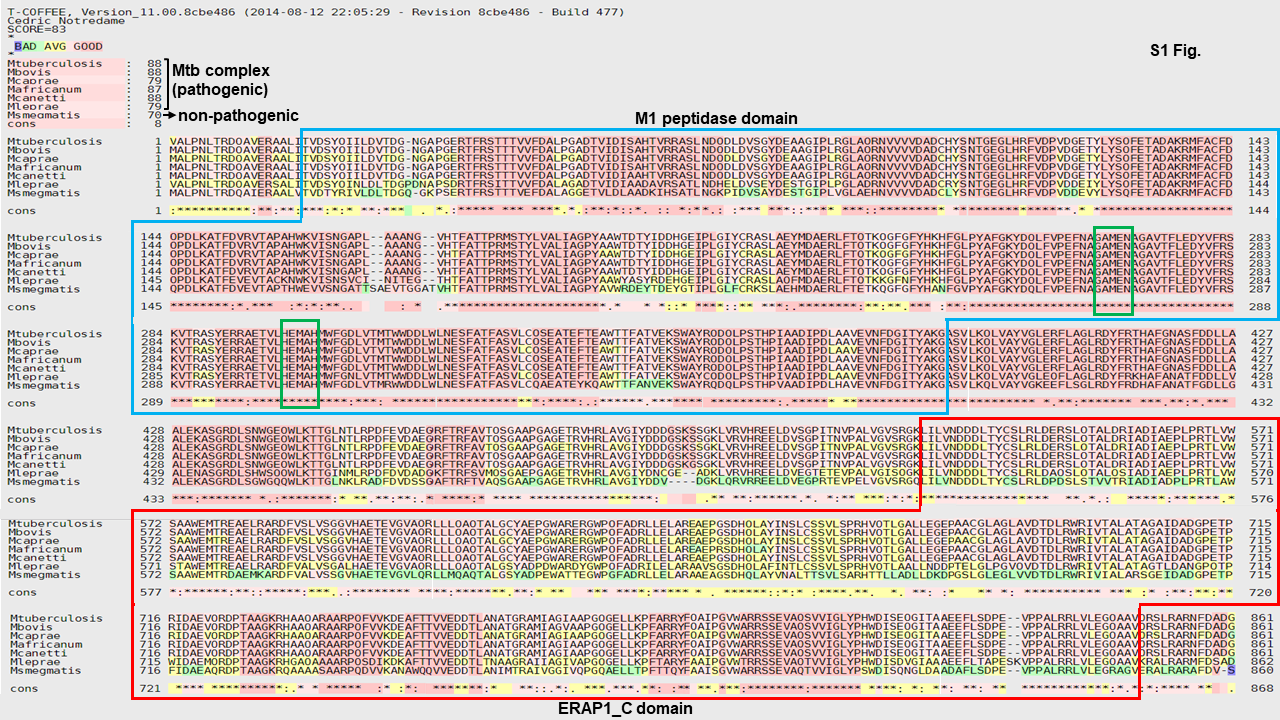

Supplement: S1 Fig — Using Expresso [32], a T-Coffee flavor that aligns multiple sequences based on structural information, PepNs from both, the slow-growing pathogenic mycobacteria (Mtb complex) viz. Mtb, M. bovis, M. caprae, M. africanum, M. canetti and M. leprae (top left) and fast growing, non-pathogenic mycobacteria representative, Msm were aligned. Expresso identified 3q7j (https://www.rcsb.org/structure/3q7j) as the reference structure, to which it aligned PepNs of Mtb complex and PepNMsm. Numbers to right of mycobacteria indicate structure-based sequence identity scores. Numbers to either end of each sequence denotes its cognate first and the last amino acid in each block. Transparent cyan box: N-terminal M1 peptidase domain; Transparent red box: ERAP1_C domain; Transparent green boxes—M1 peptidase active site (GAMEN) and zinc-binding motif (HEMAH); structure-based sequence alignment color key: Good–pink; average–yellow; poor–green; In the consensus line: cons–consensus; *—identical amino acids;. – strongly similar amino acids; .–weakly similar amino acids; () (blank space)–different amino acids. (TIF) [file pone.0215123.s005.TIF]

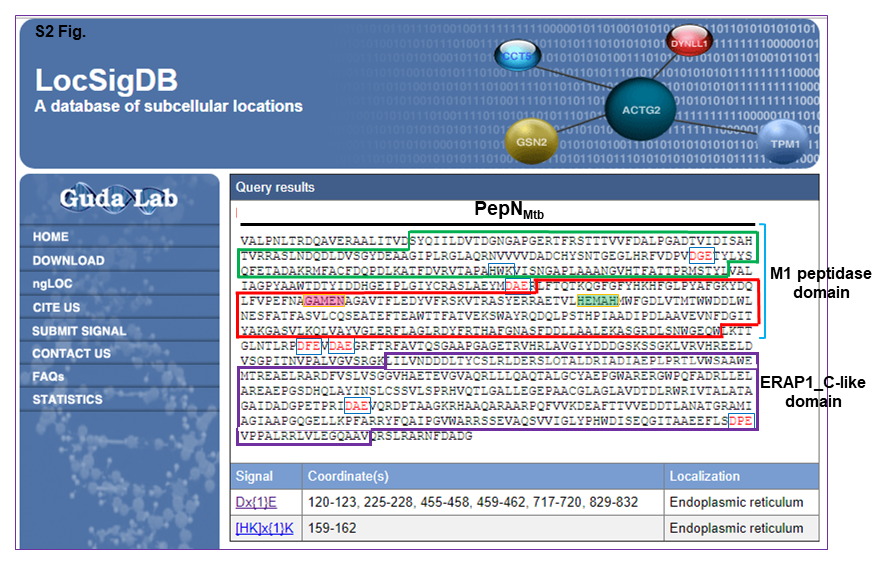

Supplement: S2 Fig — When PepNMtb is used as query sequence, LocSigDB [42] identified potential ER-homing signals (indicated in small rectangular boxes with thin blue lines, each box has three amino acids). Large transparent green and red box: M1 peptidase domain (Green—Peptidase M1 N-terminal domain; Red—Peptidase family M1 domain) and large transparent purple box: C-terminal ERAP1_C domain. The consensus signal sequences identified are at the bottom left, the co-ordinates of the identified amino acids are in the middle and the predicted localization of the query protein is to the bottom right. (TIF) [file pone.0215123.s006.TIF]

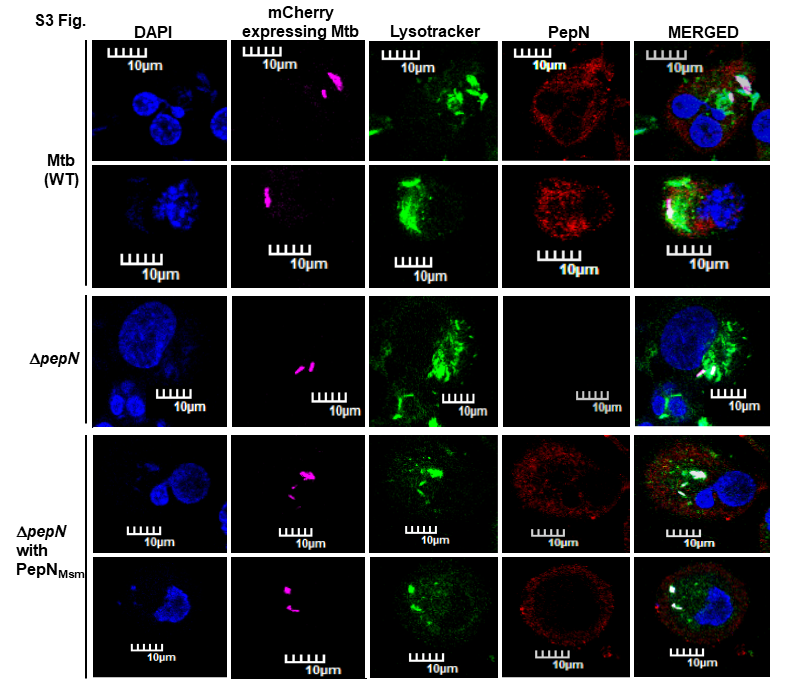

Supplement: S3 Fig — Immunofluorescence-based localization analysis (see materials and methods for protocol) of PepNMtb in THP-1 infected with either WT Mtb (top two panel rows); MtbΔpepN (middle (third) panel row) or MtbΔpepN expressing pepNMsm (bottom two panel rows). Panel columns: First: DAPI to track nuclei (blue); Second: virulent Mtb expressing mCherry (pink)—to locate infected THP-1; Third: Lysotracker green (green); Fourth: PepN (red); Final: merger of first four panels (of each row); Scale: 10 μm. Atleast 300 infected macrophages were visualized for confirming consistency of observed results. The immunofluorescence data is a representation of three independent experiments. Each independent experiment had biological duplicates. (TIF) [file pone.0215123.s007.TIF]

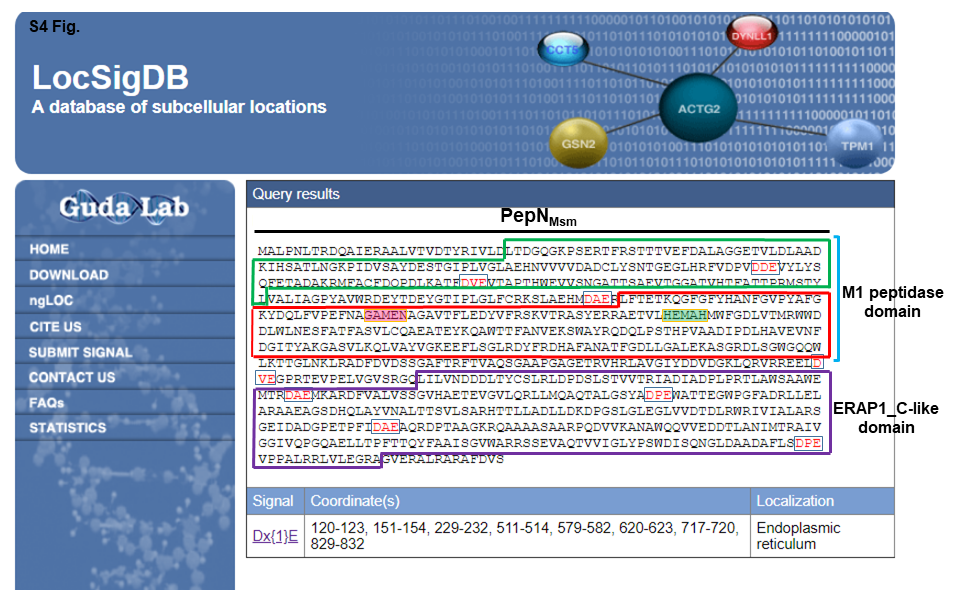

Supplement: S4 Fig — When PepNMsm is used as query sequence, LocSigDB [42] identified potential ER-homing signals (indicated in small rectangular boxes with thin blue lines, each box has three amino acids). Large transparent green and red box: M1 peptidase domain (Green—Peptidase M1 N-terminal domain; Red—Peptidase family M1 domain) and large transparent purple box: C-terminal ERAP1_C domain. The consensus signal sequences identified are at the bottom left, the co-ordinates of the identified amino acids are in the middle and the predicted localization of the query protein is to the bottom right. Unlike PepNMtb, PepNMsm lacks any [HK]x{1}K -like sequence. (TIF) [file pone.0215123.s008.TIF]

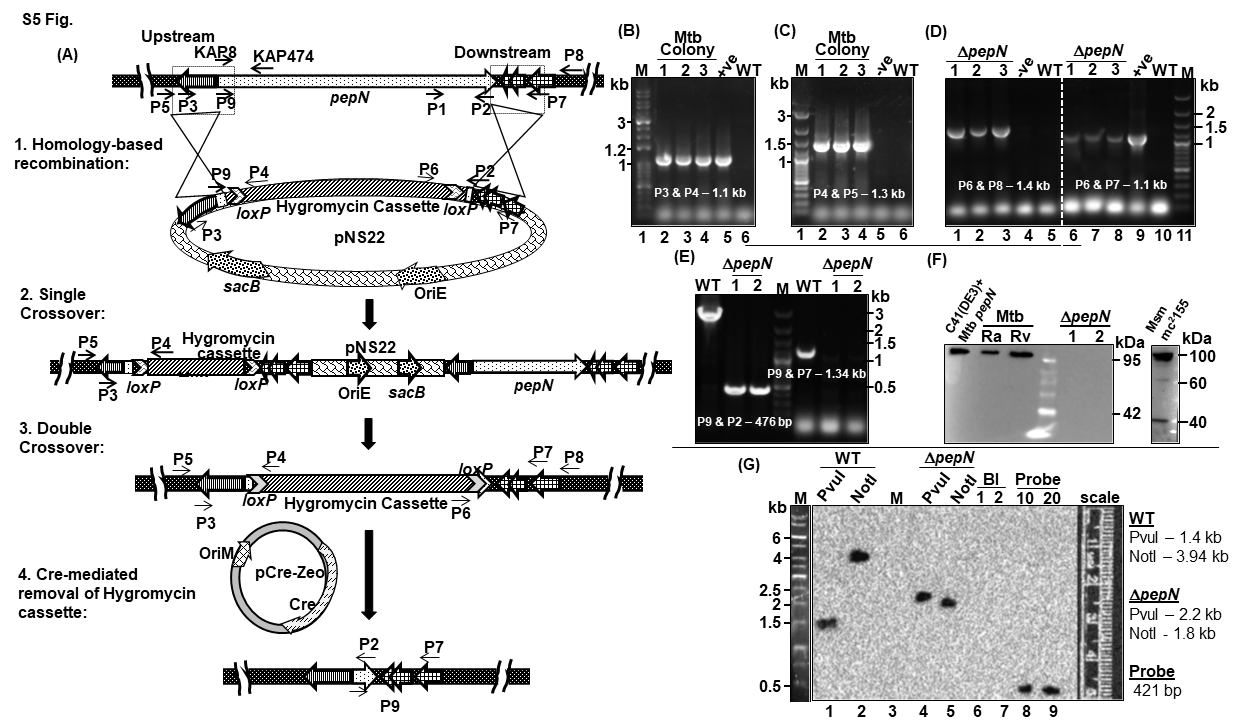

Supplement: S5 Fig — Homologous recombination-based strategy with SacB as counter selection marker [45] was adapted to generate MtbΔpepN. (A) indicates schematics of the strategy adapted. Step 1: Rectangular dotted (dull grey) boxes indicate PCR-amplified upstream and downstream regions of pepN that were cloned into the suicidal vector pKA1 to obtain pNS22. ‘X’ drawing indicates potential recombination occurring regions between pepNMtb (in the genome) and regions of pNS22. P1 to P9 –primers used for confirmation/analyses. Single line arrows below/above primers indicate forward and reverse directions. Broad arrows in pNS22 and genome indicate open reading frames. Step 2: schematics of single crossover that occurred between the upstream region to pepNMtb and pNS22 upstream fragment. Upon single crossover, the entire pNS22 is recombined into the pepNMtb locus. Step 3: schematics of pepNMtb deletion that occurred as a result of the second recombination between downstream region to pepNMtb and pNS22 downstream fragment. This led to pepNMtb being replaced by the Hygromycin resistance cassette flanked by unidirectional loxP sites. Step 4: schematics of the pepNMtb locus lacking both pepNMtb and Hygromycin resistance cassette. The resistance cassette was removed by use of pCre-Zeo encoded Cre-recombinase. (B) & (C): PCR-based validation of Step 2 (of (a)); While (B) indicates only recombination of pNS22 into the genome, (C) indicates recombination of pNS22 in to the pepNMtb upstream region. Three putative Hyg+ single crossover colonies that did not grow on 10% sucrose were selected for PCR-based single crossover screening at the upstream region (lanes 2–4) with primers P3 and P4 (B) and P4 and P5 (C). In (B): P3 and P4 primers amplify a 1.1 kb region from both the single crossovers colonies and from pNS22 (+ve: lane 5). As expected, the primers did not amplify any 1.1 kb fragment from the WT Mtb genomic DNA (lane 6). In (C): P4 and P5 primers amplify a 1.3 kb region from only the single crossov [file pone.0215123.s009.TIF]

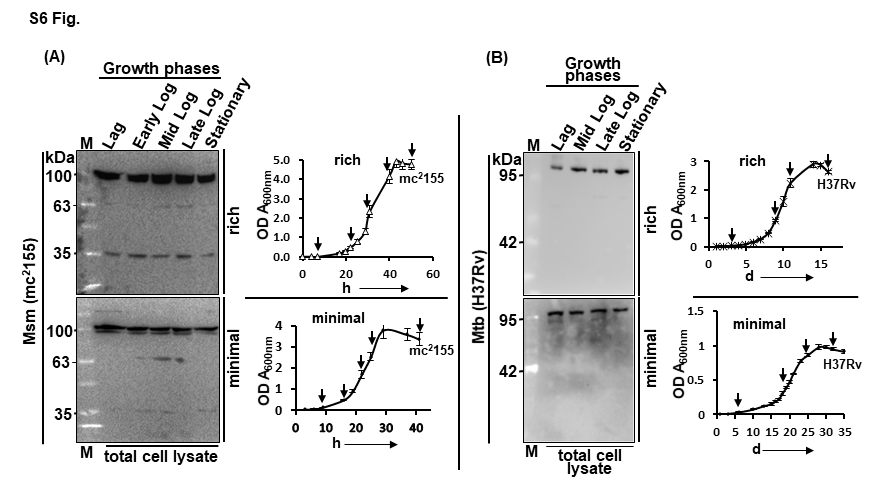

Supplement: S6 Fig — To compare steady state levels of Msm (A; mc2155) and Mtb (B; H37Rv) PepNs across different in vitro growth phases (as shown in A and B), ~250 ml cultures each were first grown in rich—(7H9 + ADC/OADC) and minimal media (Sauton’s). Then, aliquots were sampled (20 ml each), washed, lysed (by bead beating) and equal total protein (estimated by BCA kit (Thermo Fisher Scientific, USA)) resolved on 10% SDS-PAGE gels and westerns performed. Anti-PepN antibody (1:2500) was used for detecting PepN. Anti-Rabbit IgG Goat secondary antibody—1:10000; M- Protein marker. The western blots represent three independent experiments, each with biological duplicates. Optical density (A600nm) were recorded at different time-points as indicated. The data for each time point is thus mean ± SE. (TIF) [file pone.0215123.s010.TIF]

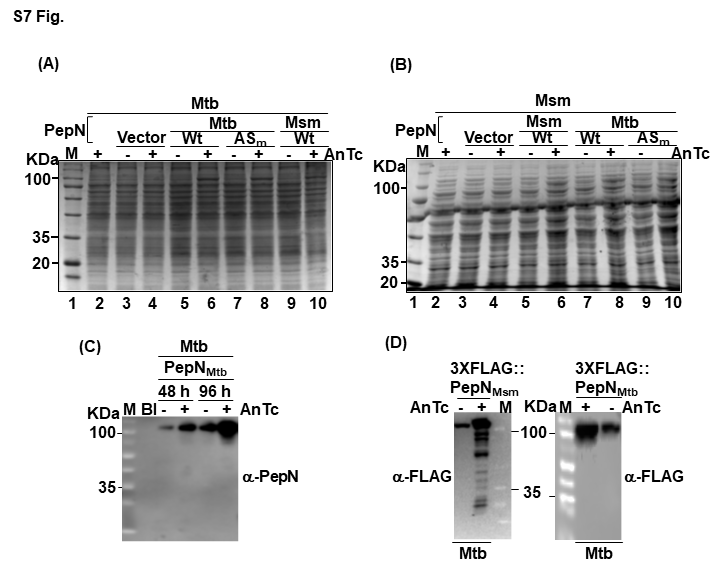

Supplement: S7 Fig — To normalize equal protein amounts (estimated by BCA kit (Thermo Fisher Scientific, USA)) for western analyses (Fig 3), total proteins of lysates of mid-log grown Mtb (A) or Msm (B) and their PepN overexpressing strains (A and B) were resolved in 10% SDS-PAGE gels and coomassie stained. (A): Lane 2—WT Mtb; Lanes 3 & 4—Mtb with vector; Lanes 5 & 6 –Mtb overexpressing PepNMtb; Lanes 7 & 8—Mtb overexpressing mutant PepNMtb; and Lanes 9 & 10 –Mtb overexpressing PepNMsm. (B): Lane 2—WT Msm; Lanes 3 & 4—Msm with vector; Lanes 5 & 6 –Msm overexpressing PepNMsm; Lanes 7 & 8 –Msm overexpressing PepNMtb; and (iv) Lanes 9 & 10 –Msm overexpressing mPepNMtb (C): Mtb tolerates over accumulation of its PepN even after 96 h of induction. Equal protein amounts from lysates of mid-log grown Mtb overexpressing PepNMtb (induced for 48 and 96 h) were resolved in 10% SDS-PAGE gels, western analysis performed and accumulating levels of PepNMtb monitored. Anti-PepNMtb antibody (1:2500) was used for detecting PepN. Anti-Rabbit IgG Goat secondary antibody—1:10000; M- Protein marker. (D): Mtb selectively proteolyzes excess 3XFLAG::PepNMsm. Equal protein amounts from lysates of mid-log grown Mtb overexpressing either 3XFLAG::PepNMsm (blot to the left) or 3XFLAG::PepNMtb (blot to the right) were loaded onto 10% SDS-PAGE gels, western analysis performed and accumulating levels of PepNMtb and PepNMsm monitored with FLAG antibody (Sigma Aldrich, USA). M—Protein marker.–and + indicate absence or presence of AnTc (100 ng/ml) respectively. Rabbit polyclonal anti-PepN antibody—(1: 2500) and anti-Rabbit IgG Goat secondary antibody (1:10000). Mouse FLAG specific monoclonal antibody–(1: 5000). (TIF) [file pone.0215123.s011.TIF]

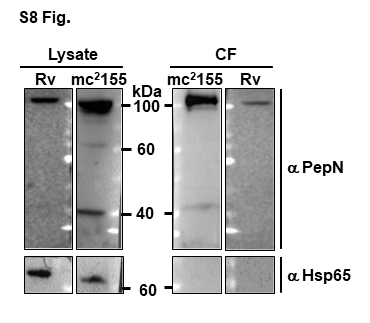

Supplement: S8 Fig — For validating secretion of PepNMtb and PepNMsm as evaluated by MS/MS (Table 1), exponential phase lab cultures of WT Msm (mc2155) and Mtb (H37Rv) were grown, cell pellets collected, lysed by beat beating, lysate filtered twice and total proteins estimated by BCA kit (Thermo Fisher Scientific, USA). The spent media were also filtered twice, TCA precipitated and total protein estimated. Equal amount of lysates (left blots) and 10-fold higher amount of culture filtrate (CF) proteins (right blots–CF) were loaded onto 10% SDS-PAGE, proteins resolved and westerns performed. Blots were developed with specific anti-PepN antibody (α PepN blots) and Hsp65-specific antibody (Abcam, UK). Hsp65 (Rv0440 in Mtb and MSMEG_0880 in Msm) is used as lysis control. Anti-PepNMtb antibody (1:2500) and anti-Rabbit IgG Goat secondary antibody (1:10000) were used for detection of PepN. Anti-Hsp65 antibody (1:2500) and anti-mouse IgG Goat secondary antibody (1:10000) were used for detection of Hsp65. White tiny bands in each blot indicate protein markers (kDa). These are representative blots of three independent experiments and their biological duplicates. (TIF) [file pone.0215123.s012.TIF]

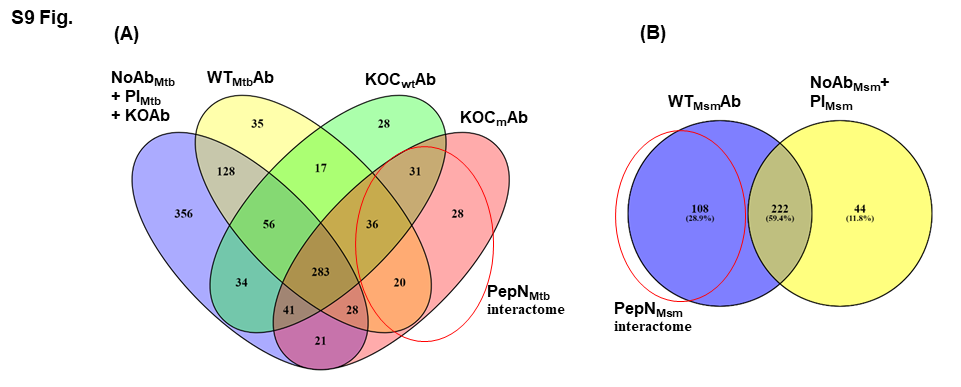

Supplement: S9 Fig — (A): Light blue oval: combined co-immunoprecipitant protein numbers of immunoprecipitated with beads alone; PIMtb—WT Mtb lysate co-immunoprecipitated with pre-immune sera; KOAb—pepNMtb KO lysate co-immunoprecipitated with plus anti-PepN antibodies. Light yellow oval: WTMtbAb—co-immunoprecipitant protein numbers obtained by co-immunoprecipitating WT Mtb lysate with anti-PepN antibodies; Light green and pink ovals: KOCwtAb and KOCmAb (respectively)—co-immunoprecipitant protein numbers obtained by co-immunoprecipitating lysates of pepNMtb KO complimented with either pepNMtb or mpepNMtb respectively, both co-immunoprecipitated with anti-PepN antibodies. Red transparent oval: indicate PepNMtb interactome protein numbers (115; S4 Table) that are found common to (i) WTMtbAb & KOCmAb (20 proteins); (ii) KOCwtAb and KOCmAb (31 proteins); (iii) WTMtbAb & KOCwtAb & KOCmAb (36 proteins) and (iv) unique to KOCmAb (28 proteins; Fig 4A; see discussion). (B): Blue circle: WTMsmAb—co-immunoprecipitant protein numbers obtained by co-immunoprecipitating WT Msm lysate with beads and anti-PepN antibodies; Yellow circle: combined co-immunoprecipitant protein numbers of NoAbMsm + PIMsm; NoAbMsm—WT Msm lysate co-immunoprecipitated with beads alone; PIMsm—WT Msm lysate co-immunoprecipitated with beads and pre-immune sera. (A and B): Protein lists from each group were fed into Venny (http://bioinfogp.cnb.csic.es/tools/venny/index2.0.2.html), venn diagrams generated and common and unique proteins identified. (TIF) [file pone.0215123.s013.TIF]
